# Use of biogenic silver nanoparticles in enhancing shelf life of *Morus alba* L. at post harvest stage

**DOI:** 10.1038/s41598-020-65953-7

**Published:** 2020-06-02

**Authors:** Dipayan Das, Palash Mandal

**Affiliations:** 1 Senior Research Scholar, CSIR-UGC NET JRF, Plant Physiology and Pharmacognosy Research Laboratory, Department of Botany, University of North Bengal, Raja Rammohanpur, Siliguri, West Bengal 734013 India; 2 Assistant Professor, Department of Botany, University of North Bengal, Raja Rammohanpur, Siliguri, West Bengal 734013 India

**Keywords:** Biochemistry, Plant sciences, Nanoscience and technology

## Abstract

*Morus alba* is one of the most important cultivated crop in Indian sub-continent contributing towards production of silk fibre that carries economic importance worldwide. Post harvest preservation of *M. alba* leaves is a challenging factor as decrease in concentration of essential metabolites that needed for silk gland development takes place. Decrease in chlorophyll, protein, sugar concentration and increase in accumulation of free radicals and ROS takes place at post harvest stage of preservation, putting negative impact on larval development indicated by high mortality rate. Silver nitrate and nanosilver solution acts as an effective preservative, enhances the activity of enzymatic and non-enzymatic antioxidants thereby reducing the harmful effect of accumulated free radicals and ROS. The effectiveness of nanosilver solution was found to be on the upper site without any significant difference than silver nitrate, as higher retention of primary metabolites like pigments, proteins, and sugar takes place. The impact of feeding nanosilver preserved leaves on silkworm was found on the positive trend as larval growth rate, cocoon weight, shell weight, effective rate of rearing was observed almost same to the larvae fed with fresh leaves.

## Introduction

*Morus alba* an economically important crop, placed by Bentham and Hooker under the family Moraceae. *M. alba* leaves are used for feeding monophagous insect silkworm, which produces raw silk contributing towards world economy. Development of silk gland and production of quality silk depends upon leaf protein content. Indeed larvae act as an intermediate agent converting leaf protein into silk fibre^1^. Deviation in leaves nutrient quality puts significant impact over larval growth and cocoon formation^2^.

Silk industry was an agro-based labour intensive industry which generates high employment among rural peoples^3^. Practice of silkworm rearing consists of two important activities: (1) cultivation of *M. alba*, for fresh leaves and (2) rearing of silkworm. Rearing is an indoor practice while cultivation of *M. alba* leaves is an outdoor practice, requiring an open land. Thus rearing practice remains restricted to those farmers those bears marginal to small scale lands^4^. Landless farmers generally migrate from one garden to another or move to urban areas in search of work leaving the traditional practice. Some farmers even purchase leaves from others gardens^4^ but regular purchasing of leaves and its carrying cost increases the overall cost of production. Preservation by retaining the leaf quality on purchasing the leaves once in a while may serve as a solution to this problem.

Wilting, discolouration, senescence, high respiration rate, decay and microbial growth are the main cause which limits postharvest extension of shelf life. Fresh leaves are live and are subjected to environmental and physiological variables leading to changes in preharvest and postharvest quality^5,6^. Microbial proliferation causes rapid senescence and degradation of macromolecules by rising ROS and free radical percentage^7^. Decolouration by chlorophyll degradation resulting in yellowing of leaves is the most conspicuous indicative phenomenon of leaf senescence^7^. It has been reported that many internal and external factors are generally involved in leaf chlorophyll preservation^8,9^. In presence of light, leaf chloroplast was the major site for ROS generation and during senescence disassembly of photosynthetic apparatus causes disturbance of reducing equivalents of electron chain resulting enhancement in ROS accumulation^10^. Excessive ROS accumulation causes cellular damage, evident by degradation of pigment, proteins, lipids, carbohydrates and even nucleic acid^11^. To overcome oxidative injury, plant causes detoxification of excess generated ROS by activating enzymatic and non-enzymatic enzymological activities^12^. ROS scavenging activity was triggered by the activity of the enzymes like superoxide dismutase (SOD), catalase (CAT), glutathione peroxidase (GPOX), ascorbate peroxidase (APX) which works in a coordinated fashion converting superoxide (O_2_^•−^) to hydrogen peroxide (H_2_O_2_) and finally to H_2_O^13,14^. Glutathione, ascorbic acid, carotenoids are mainly involved in non-enzymatic antioxidant activities. Glutathione helps in the generation of ascorbic acid which detoxify O_2_^•−^ inside chloroplast^15^, whereas carotenoids protects photosynthetic apparatus from stress mediated damages^16^. Phenolics are also classified as non-enzymatic antioxidants which nullifies the toxic effect of free radicals^17^.

With implementation of stress, auto-activation of defensive enzymatic and non-enzymatic molecules takes place. But at post harvest stage with increase in days of preservation these defensive activities decreases. Presence of elicitor or preservative may prolong the shelf life by up-regulating the defensive pathways. Silver nitrate (SN) was the most applied silver salt as preservative in the field of horticultural crop, as they inhibit microbial proliferation, preventing vascular occlusion^18^. The impact of SN in prolonging vase life of rose^19^, tuberose^20^ has been well documented. It has been reported that there might be the possibility of SN causing toxicity to living organism^21^, putting a backward thrust towards its application. Nanosilver (NS) may serve as an alternate option as it has lowest toxic effect than any other silver forms^22^. In comparison to ionic form of silver, nanosilver have highly developed surface area making it more reactive, besides this its physico-chemical property allows to interact with living cell differentially^23^. Nanosilver at low concentration may act as an effective preservative which not only prevents microbial growth^24,25^, maintaining xylem integrity but also activates protective enzymological activities^26^. Implementation of NS as preservatives has been reported to extend the shelf life of *Dianthus*^27^, *Chrysanthemum*^28^, tulip^29^,  *Gerbera*^30^. However most of the preservative aspects of SN and NS have been reported to extend shelf life of economically important flowering twigs, almost no report was obtained that describes their ability to preserve postharvest leaf samples.

Current study was conducted assuming the hypothesis that biosynthesized silver nanoparticles will bear the ability to extend the shelf life of S1 cultivar of *M. alba* leaves at postharvest stage. In the current study three attempts were made, first to investigate the effect of NS as preservative solution, in extending shelf life of *M. alba* leaves by retaining valuable metabolite concentration; secondly a comparative aspect of two preservative solutions, NS and SN; and lastly whether there is any adverse effect of feeding preserved leaves on silkworm rearing system.

## Results

### Synthesis of silver nanoparticles and its characterization

**Figure 1:**
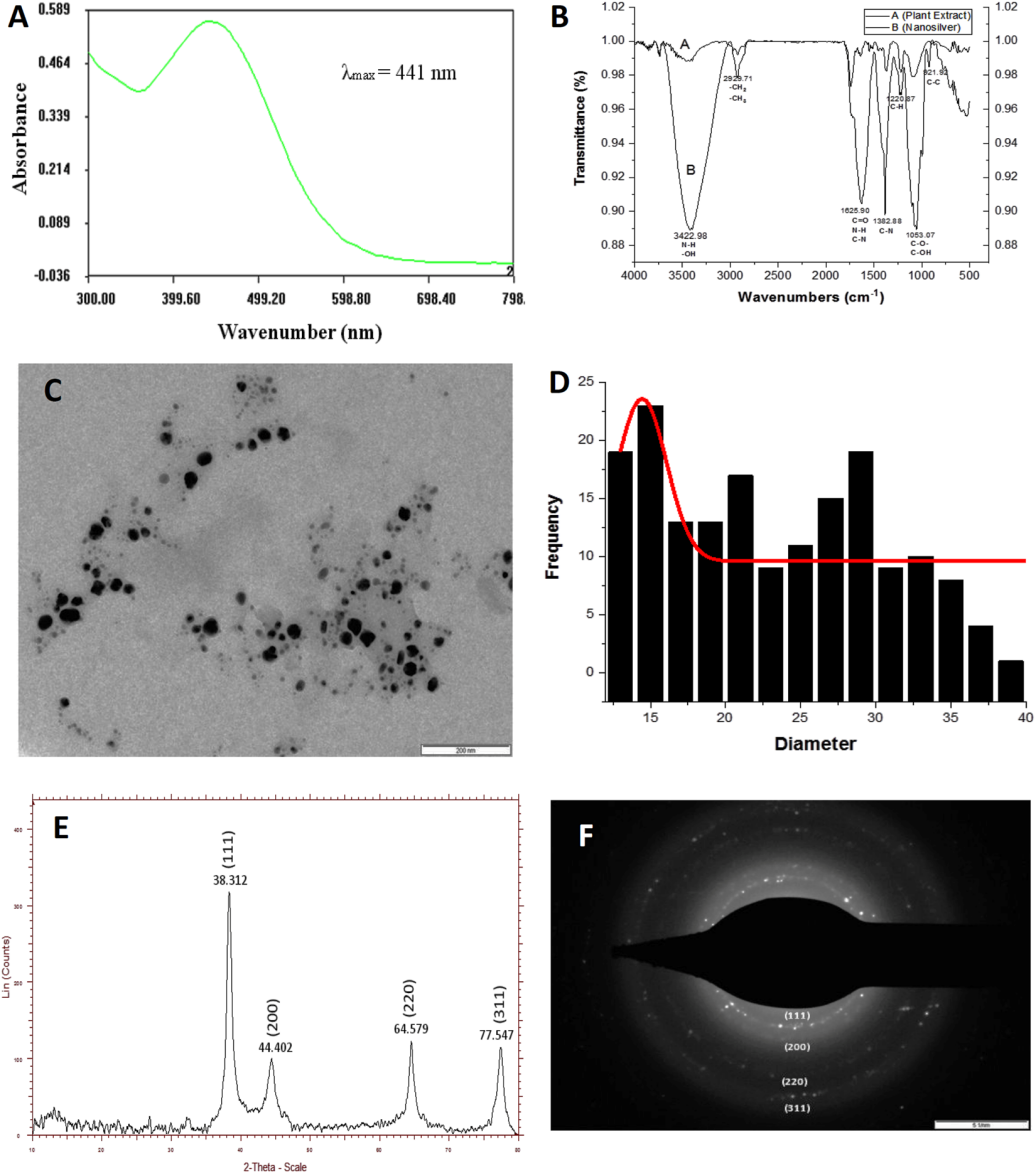
Characterization of phyto-synthesized silver nanoparticles through (**A**) UV-Visible spectrophotometer, (**B**) fourier transform infrared spectroscopy, (**C**, **D**) transmission electron microscopy and size distribution, (**E**) X-ray diffraction analysis, and (**F**) selected area electron diffraction.

Initial confirmation of nanosilver formation was obtained by observing the colour change of the solution from transparent to blackish brown. Confirmatory validation was done through UV–Visible spectrophotometer which showed plasmon peak at ~441 nm. Synthesized nanoparticles were spherical in shape with size distribution range from 12–39 nm, as revealed by TEM analysis. FT-IR spectra showed almost similar peak positions of biosynthesized nanosilver and plant extract used for biosynthesis. The FT-IR absorption peak at 3422.98, 2929.71, 1625.90, 1382.88, 1053.07 and 921.92 cm^−1^ corresponds to –OH stress of phenols and alcohols and N–H vibrational stretching, –CH_2_ and –CH_3_ vibration, C–N vibration of amide I band, C-N vibrational stress, C–O– and C–OH vibration of protein and carbohydrate, and C–C bond of branch alkanes respectively. Four major 2θ peaks at 38.312°, 44.402°, 64.579°, 77.547° were obtained from X-ray diffraction pattern which corresponds to (hkl) values of (111), (200), (220), (311) of Bragg’s reflection plane. Electron diffraction (SAED) pattern showed bright diffraction spots of hkl value corresponds to (111), (200), (220), and (311) (Fig. 1).

### Changes in primary metabolites and proline content

**Figure 2:**
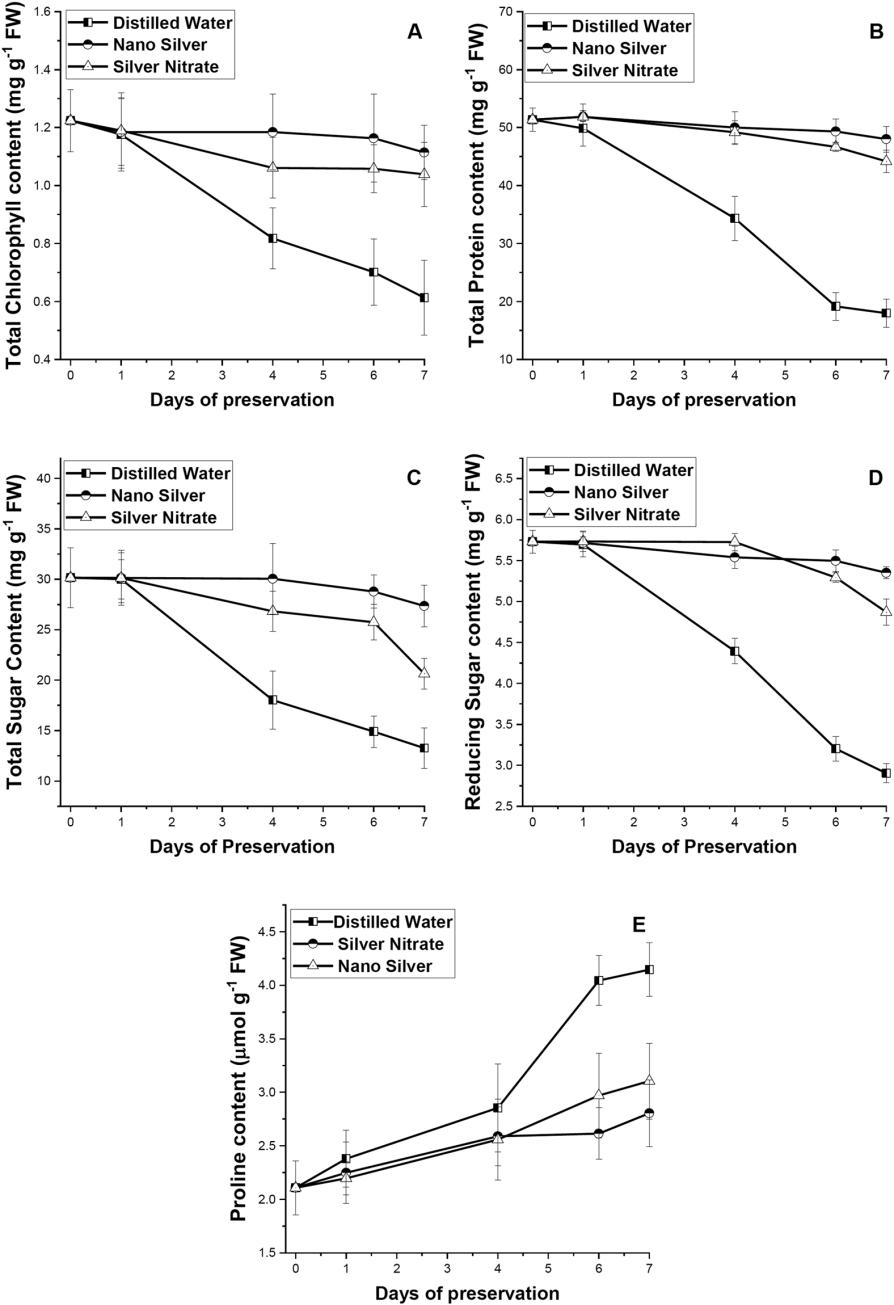
Effect of preservation of S1 genotype of *M. alba* leaves with distilled water, nanosilver and silver nitrate on (**A**) total chlorophyll, (**B**) total protein, (**C,D**) total and reducing sugar, and (**E**) proline content. Effect of preservative solutions on *M. alba* leaves was observed at regular interval of 0D, 1D, 4D, 6D and 7D. The results were expressed as Mean ± SDEV, n = 3.

With increase in days of preservation, the concentration of total chlorophyll, total soluble protein, total and reducing sugar decreases and that of free proline content increases (Fig. 2). In distilled water set a greater increase in proline content and greater decrease in content of total soluble protein and other primary metabolites was observed. Leaves preserved in SN and NS solution showed a lower magnitude of increase in free proline content and less depletion in soluble protein and other primary metabolites. Leaves preserved in NS solution exhibited greater retention of primary metabolites than distilled water set (p ≤ 0.05).

### Changes in reactive oxygen species and MDA content

**Figure 3:**
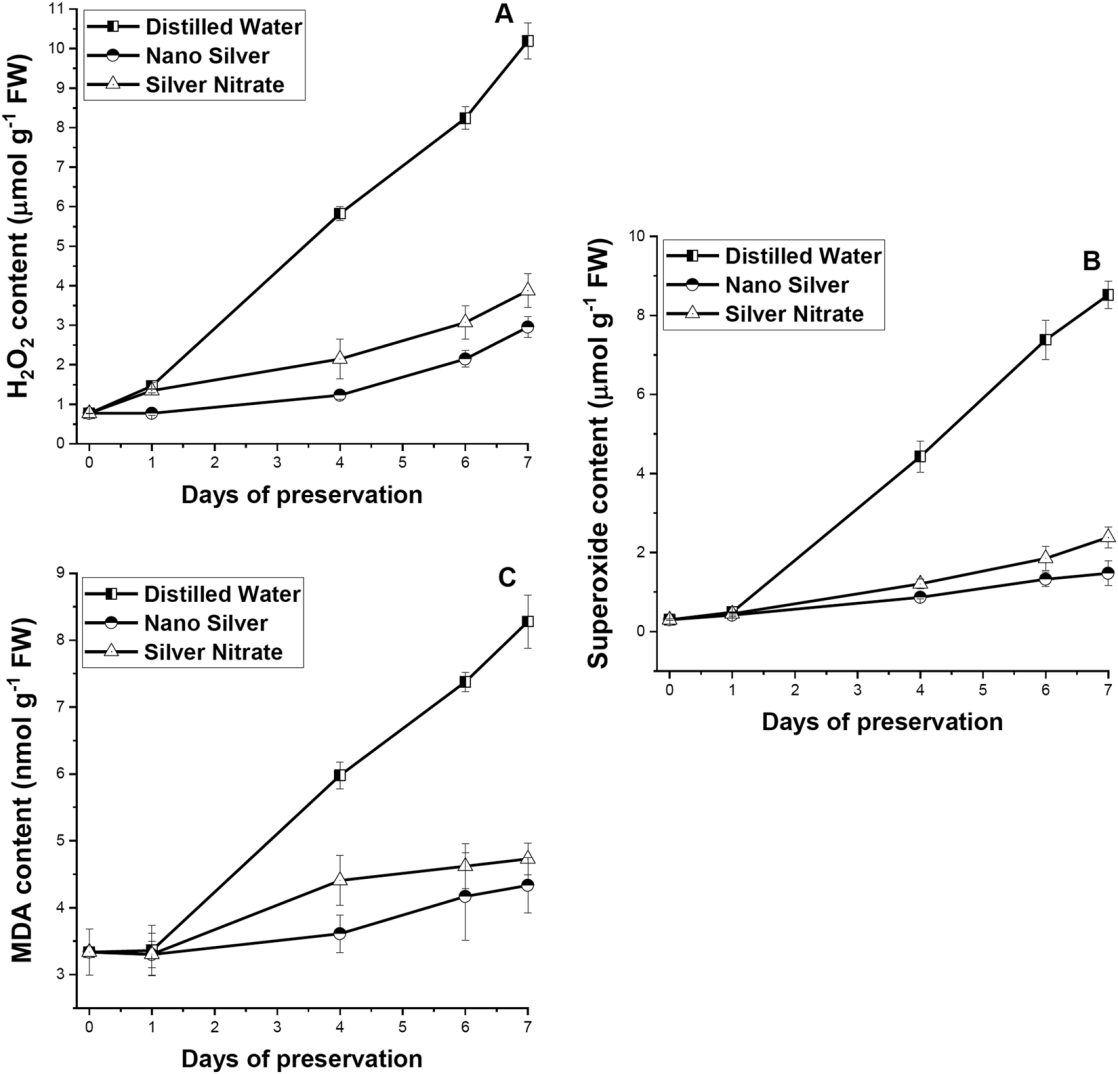
Effect of preservation of S1 genotype of *M. alba* leaves with distilled water, nanosilver and silver nitrate on (**A**) hydrogen peroxide, (**B**) superoxide, and (**C**) malondialdehyde content. Effect of preservative solutions on stress content of *M. alba* leaves was observed at regular interval of 0D, 1D, 4D, 6D and 7D. The results were expressed as Mean ± SDEV, n = 3.

**Figure 4:**
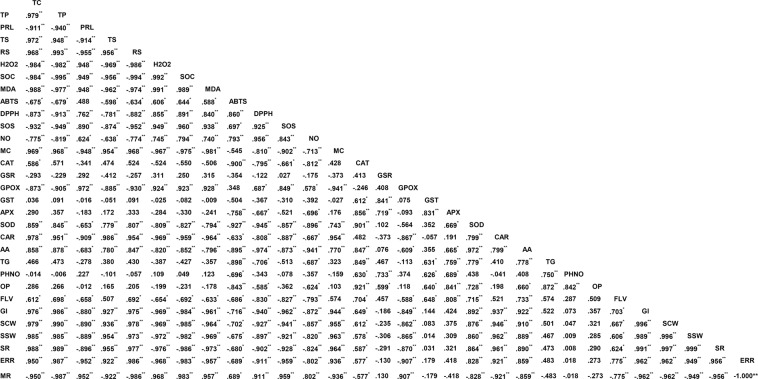
Two tailed Pearson correlation representing interrelationship among the post-preservative quality detecting parameters and rearing parameters. Significance level was indicated by “**” and “*” indicating correlation was significant at the 0.01 and 0.05 level (2-tailed) respectively. The abbreviation used means the following: TC= total chlorophyll, TP=total protein, PRL= proline, TS= total sugar, RS= reducing sugar, H2O2= hydrogen peroxide, SOC= superoxide content, MDA= malondialdehyde, SOS= superoxide scavenging, NO= nitricoxide, MC= metal chelating, CAT= catalase, GSR= glutathione disulfide reductase, GPOX= glutathione peroxidase, GST= glutathione S-transferase, APX= ascorbate peroxidase, SOD= superoxide dismutase, CAR= carotenoids, AA= ascorbic acid, TG= total glutathione content, PHNO= phenol, OP= ortho-phenol, FLV= flavonoid, GI= growth index, SCW= single cocoon weight, SSW= single shell weight, SR= shell ratio, ERR= effective rate of rearing, MR= mortality rate.

The contents of H_2_O_2_, O_2_^•−^, and MDA increased rapidly in distilled water set than in leaves preserved in NS and SN solution (Fig. 3). The magnitude of increase of these substances in leaves preserved in NS solution showed significant negative difference in comparison to distilled water set (p ≤ 0.05). Overall increase in ROS and MDA content was higher in leaves preserved in SN solution than NS solution but lesser than distilled water without any significant difference (p ≤ 0.05) except for H_2_O_2_, which exhibited large significant (p ≤ 0.05) increase in distilled water set. Increase in ROS and MDA content with increase in days of preservation bears negative correlation with primary metabolite content (p ≤ 0.01) and positive correlation with proline and antioxidant activity (p ≤ 0.01 and 0.05) (Fig. 4).

### Changes in free radical scavenging activities, and metal chelating activity

**Figure 5:**
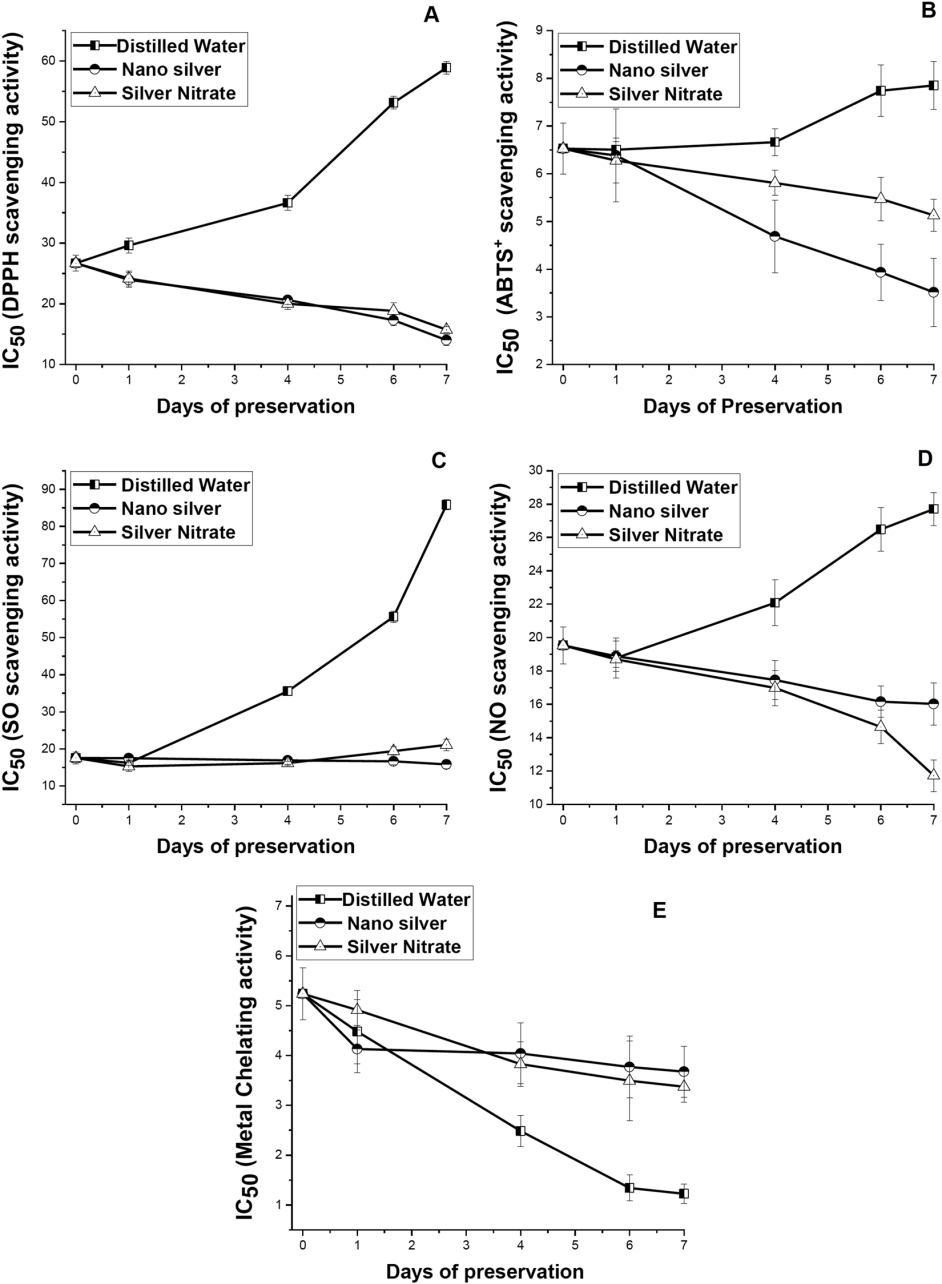
Effect of preservation of S1 genotype of *M. alba* leaves with distilled water, nanosilver and silver nitrate on (**A**) diphenyl-1-picrylhydrazyl, (**B**) 2,2-azino-bis 3-ethylbenzthiazoline-6-sulphonic acid, (**C**) superoxide, (**D**) nitric oxide, and (**E**) metal chelating activity. Effect of preservative solutions on antioxidant activity of *M. alba* leaves was observed at regular interval of 0D, 1D, 4D, 6D and 7D. Smaller the IC50 value more was the antioxidant activity. The results were expressed as Mean ± SDEV, n = 3.

With increase in days of preservation free radical scavenging activity increased in leaves preserved with NS and SN solution as revealed by decreased in IC_50_ values, while reverse trend was observed in distilled water set (Fig. 5). In distilled water set high magnitude of decrease in DPPH and SO scavenging activity was observed from 4DS onwards. Changes in DPPH scavenging activity with increase in days of preservation in NS and SN solution differs significantly with the observed changes in distilled water set (p ≤ 0.05). Significant difference was also observed for ABTS scavenging activity between leaves preserved in NS solution as compared to distilled water preservation (p ≤ 0.05). The correlation matrix showed negative significant correlation (p ≤ 0.01 and 0.05) between free radical scavenging activity and primary metabolite content, but the correlation was actually positive because free radical scavenging activity was expressed in terms of IC_50_. Leaves preserved in distilled water showed greater metal chelating activity than leaves preserved in NS and SN solution without any significant difference.

### Changes in enzymatic antioxidant activities

**Figure 6:**
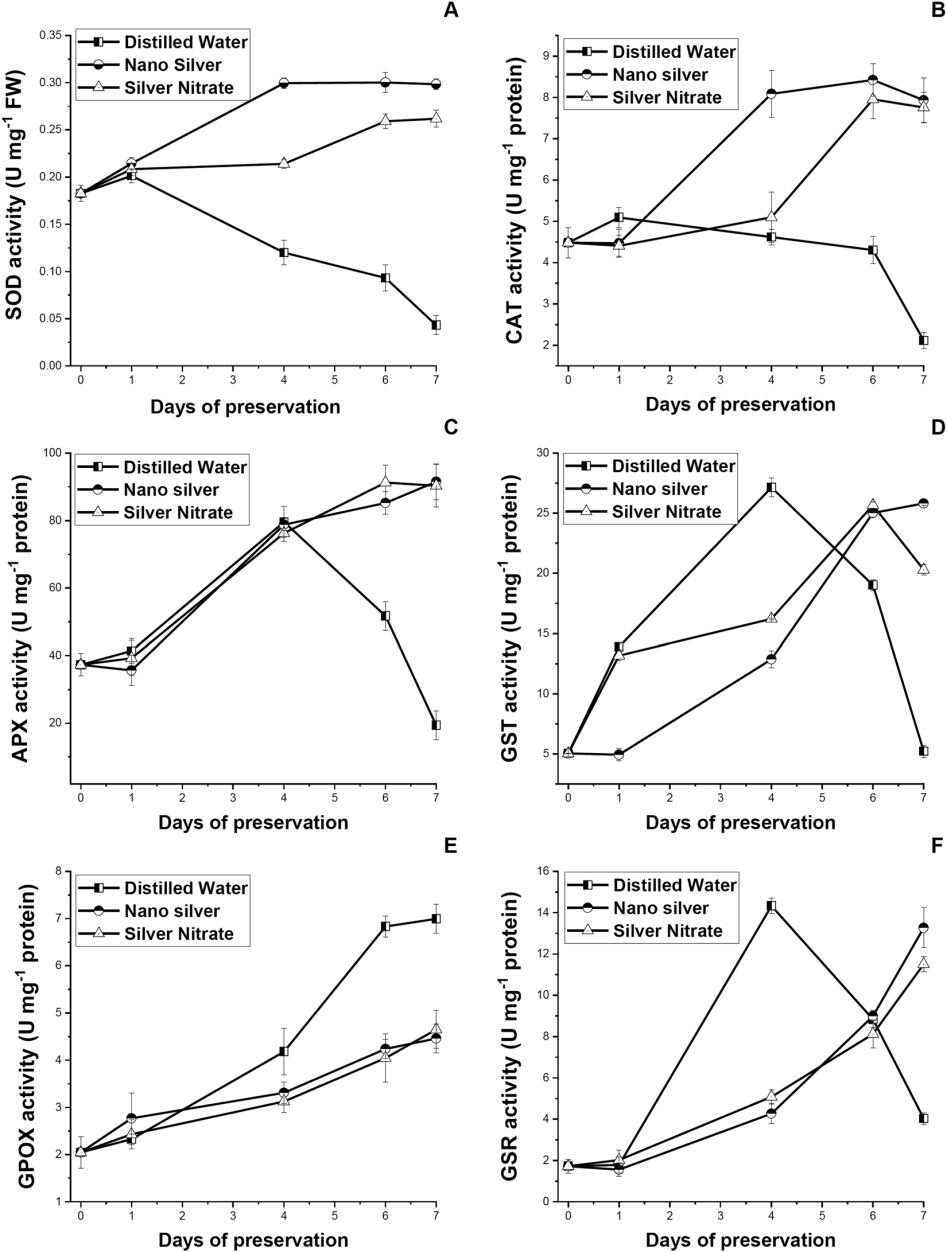
Effect of preservation of S1 genotype of *M. alba* leaves with distilled water, nanosilver and silver nitrate on (**A**) superoxide dismutase, (**B**) catalase, (**C**) ascorbate peroxidase, (**D**) glutathione S-transferase, (**E**) glutathione peroxidase, and (**F**) glutathione disulfide reductase content. Effect of preservative solutions on antioxidant enzymatic activity of *M. alba* leaves was observed at regular interval of 0D, 1D, 4D, 6D and 7D. Larger the content, greater was the defensive activity, less was the stress accumulation. The results were expressed as Mean ± SDEV, n = 3.

As days of preservation increases, an escalating trend in enzymatic antioxidant activity was observed (Fig. 6). A rapid increase in GSR, GST, and APX activity was observed in distilled water set up to 4DS, after which gradual decrease in activity takes place, except for GPOX which maintained a linear increasing trend till last day. While SOD activity showed declining trend from day one of preservation. Antioxidant enzymatic activity of leaves preserved in NS and SN solution increases gradually, reaching a maximum at 6DS, after which drop in activity in CAT, and GST was observed for SN. Broadly, antioxidant enzymatic activity in leaves preserved in NS and SN solution increases with days of preservation without any significant difference (p ≤ 0.05) between the two preservatives. Correlation matrix reveals that with increase in concentration of secondary metabolites, increase in enzymatic antioxidant activity takes place (p ≤ 0.01 and 0.05).

### Changes in non-enzymatic antioxidant activities

**Figure 7:**
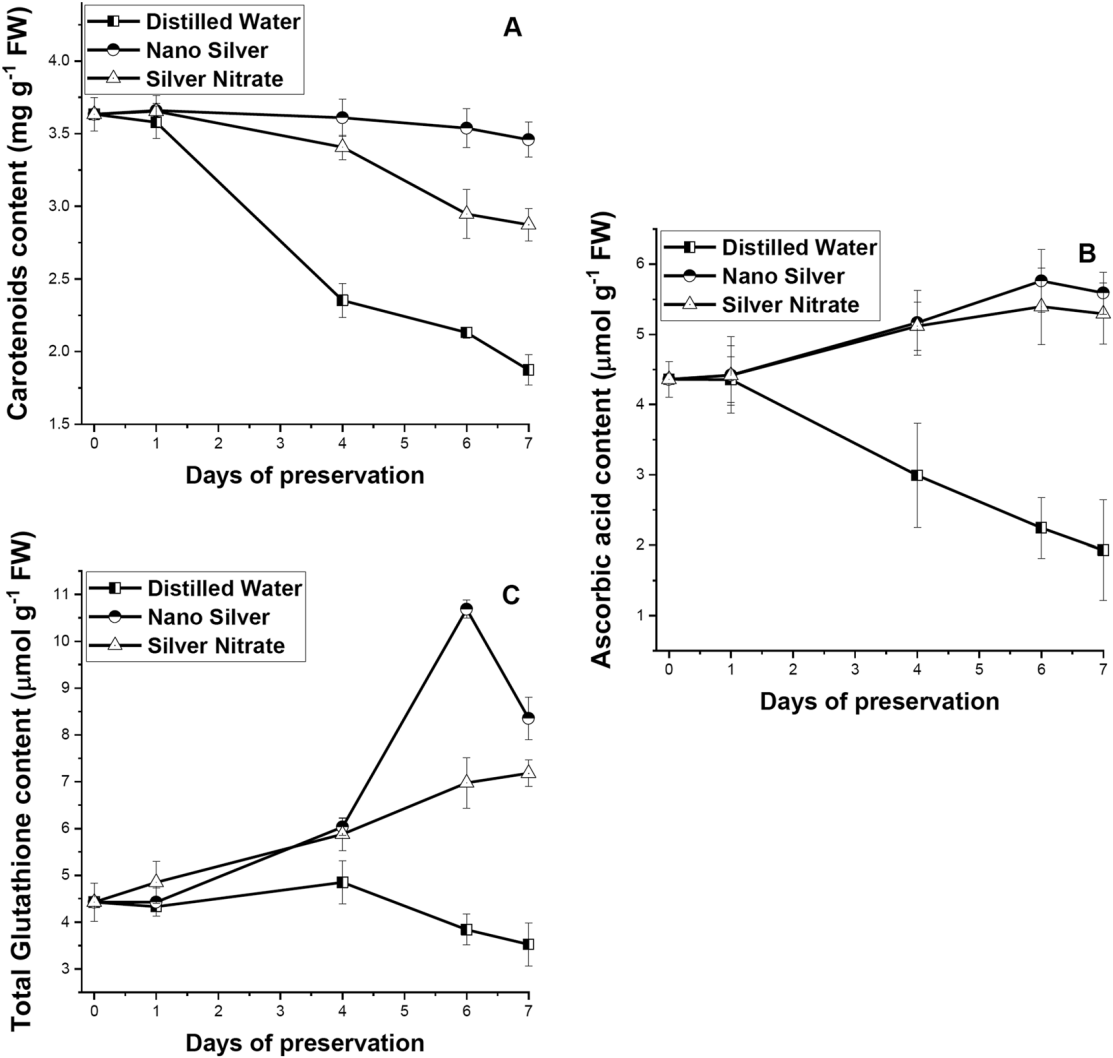
Effect of preservation of S1 genotype of *M. alba* leaves with distilled water, nanosilver and silver nitrate on (**A**) carotenoids, (**B**) ascorbic acid, and (**C**) total glutathione content. Effect of preservative solutions on non-enzymatic antioxidant activity of *M. alba* leaves was observed at regular interval of 0D, 1D, 4D, 6D and 7D. The results were expressed as Mean ± SDEV, n = 3.

An increase in content of total glutathione was observed from early days, in leaves preserved in NS, reaching a maximum on 6DS, after which rapid decline takes place (Fig. 7). In distilled water set decrease in content of total glutathione was observed after 4DS. Leaves in NS and SN solution showed significant (p ≤ 0.05) higher ascorbic acid content than distilled water set, where decrease content takes place from 1DS of preservation. Decrease in carotenoids content was observed from 4DS onwards in all the preservative solutions, with maximum magnitude of decrease in distilled water followed by SN. Leaves preserved in NS showed significant (p ≤ 0.05) retention of carotenoids with increase in days of preservation in relation to distilled water preservation. Increase in ascorbic acid activity in preserved leaves plays a significant (p ≤ 0.01) role in retention of chlorophyll, protein, and sugar content as revealed from correlation matrix.

### Changes in polyphenol contents

**Figure 8:**
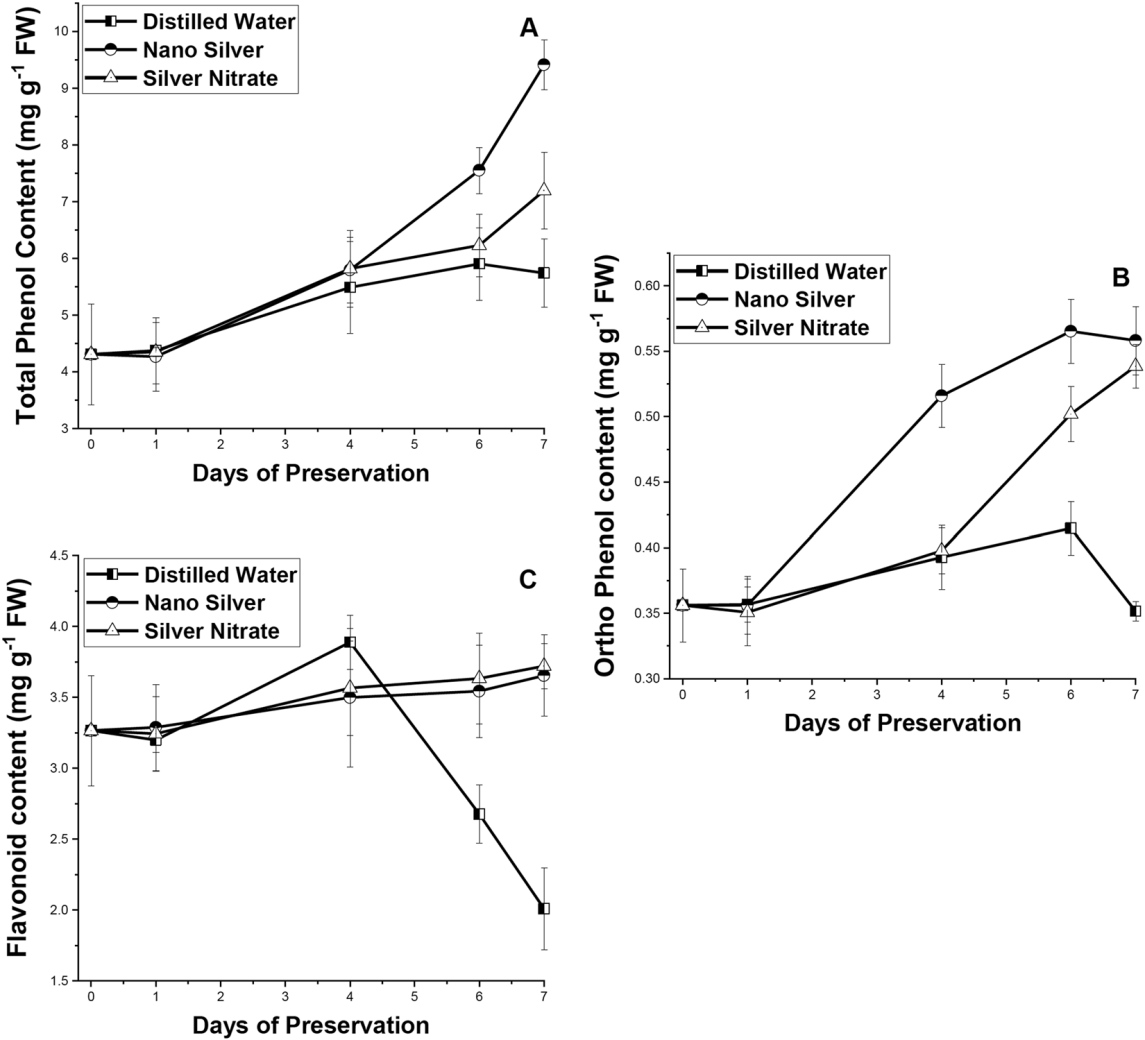
Effect of preservation of S1 genotype of *M. alba* leaves with distilled water, nanosilver and silver nitrate on (**A**) total phenol, (**B**) ortho-dihydric phenol, and (**C**) flavonoid content. Effect of preservative solutions on secondary metabolite content of *M. alba* leaves was observed at regular interval of 0D, 1D, 4D, 6D and 7D. The results were expressed as Mean ± SDEV, n = 3.

The concentration of polyphenol increases with increase in days of preservation (Fig. 8). Significant difference (p ≤ 0.05) in phenol content was observed between initial and final day of preservation. In NS and SN set increase in phenol content was observed till last day, with greater increase in NS than SN, while in distilled water set decrease in content was observed on the last day. Almost similar trend of changes was noticed for ortho-dihydric phenol content without any significant difference (p ≤ 0.05) between preservative solutions. In distilled water set a greater increase in flavonoid content takes place till 4DS, after which a greater decrease was noted, while in NS and SN set increasing trend was observed till last day without any significant difference (p ≤ 0.05).

### Changes in rearing data collection

**Figure 9:**
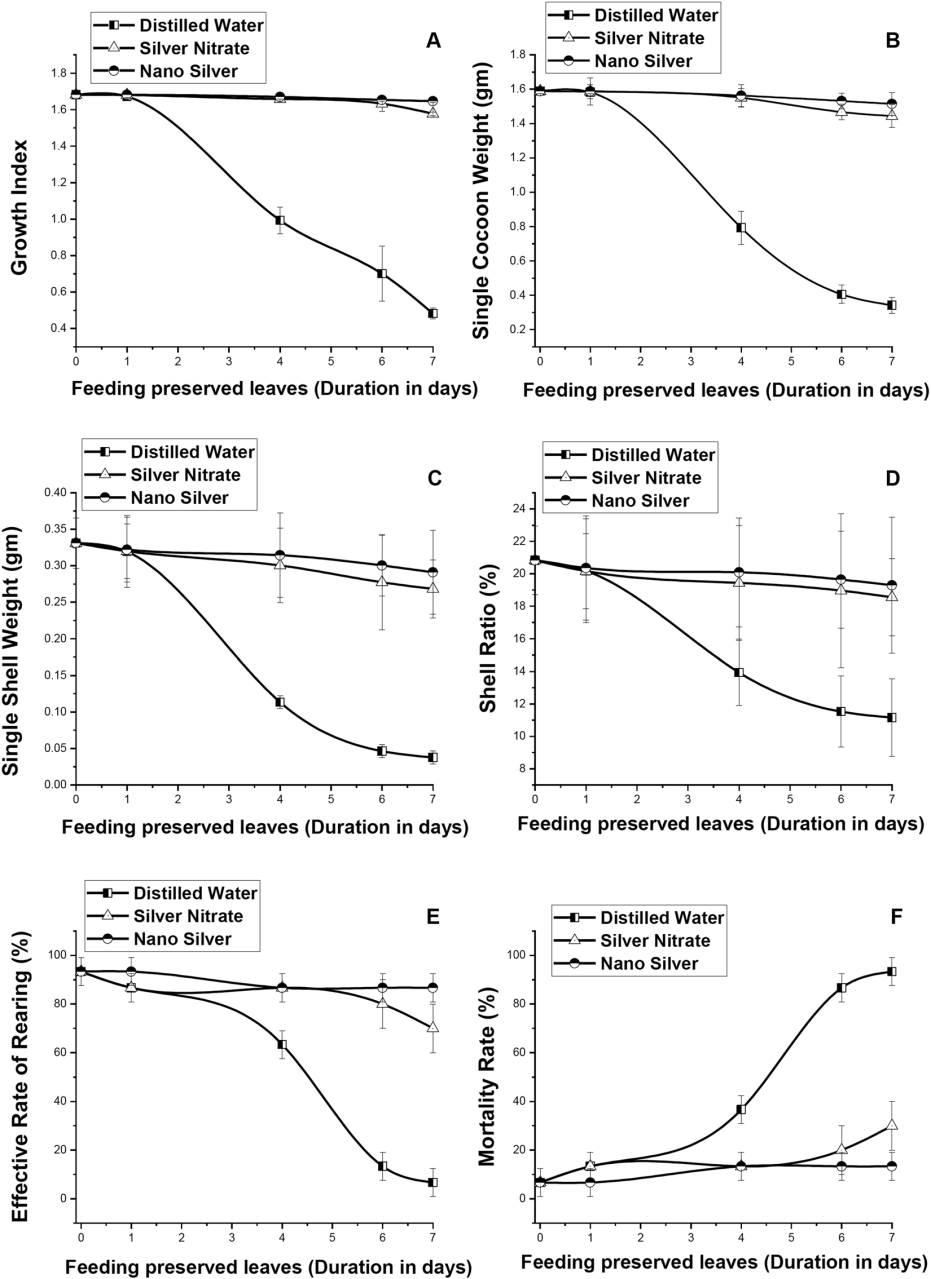
Effect of feeding S1 genotype of *M. alba* leaves, preserved with distilled water, nanosilver and silver nitrate on (**A**) growth index, (**B**) single cocoon weight, (**C**) single shell weight, (**D**) shell ratio, (**E**) effective rearing rate, and (**F**) mortality rate. Silkworm larvae were fed with leaves preserved for 0D, 1D, 4D, 6D and 7D. The results were expressed as Mean ± SDEV, n = 3.

A significant decrease in growth index was observed when fifth instar larvae was fed with leaves preserved in distilled water (Fig. 9). Decrease in GI reaches up to ~71% when fed with 7DS preserved leaves. On feeding leaves preserved with NS and SN decrease in GI was negligible in comparison with fresh leaves, with maximum decrease takes place on feeding 7DS preserved leaves which was ~2% and ~6% respectively. Significant (p ≤ 0.05) difference was observed on comparing GI of larvae fed with leaves preserved in NS and distilled water. It was observed from correlation analysis that greater the retention of primary metabolite (p ≤ 0.01) along with lesser the accumulation of free radicals (p ≤ 0.01) in leaves better was the GI.

After the larvae stops spinning, a significant decrease in SCW and SSW was observed from the larvae fed with 4DS, 6DS and 7DS distilled water preserved leaves, with maximum decrease on feeding 7DS preserved leaves ~78% and ~88% respectively. Larvae fed with leaves preserved in NS and SN showed significant (p ≤ 0.05) retention of SCW and SSW. More over larvae fed with leaves preserved in NS solution showed higher retention of SCW and SSW than larvae fed with leaves preserved in SN solution. Significant (p ≤ 0.01) positive relationship was observed between the concentration of chlorophyll, protein, sugar present in the leaves and cocoon produced by the larvae. Shell ratio of larvae fed with leaves preserved in NS solution was observed to be more elevated than larvae fed with leaves preserved in SN and distilled water solution.

The ERR of larvae depends on the amount of stress imposed on leaves with increase in days of preservation in different preservative solutions. It was observed that reduction in ERR was less in larvae fed with leaves preserved in NS solution, with maximum reduction of ~7%. Consequently larvae fed with leaves preserved in distilled water and SN solution showed maximum reduction of 92% and 24% respectively.

A gradual increase in mortality rate was observed on feeding larvae with leaves preserved with distilled water, with maximum mortality of ~93% on feeding with 7DS leaves. Consequently larvae fed with leaves preserved in NS and SN solution showed high survival rate, with maximum mortality of ~13% and ~30% respectively on feeding 7DS preserved leaves.

## Discussion

The prime challenge faced during leaf preservation was the gradual appearance of senescence with increase in days of preservation. Senescence was primarily characterized by breakdown of photosynthetic pigments resulting in gradual reduction in photosynthetic rate and photosynthesis: respiration ratio^31^. Beside this, differences which were observed on feeding senescence leaves include low growth index, decrease in cocoon and shell weight, and high larval mortality. Several senescence retardation mechanisms were adopted by the leaves and it’s up and down regulation in presence of silver nitrate and nanosilver was evaluated for determining their efficacy. In presence of silver nitrate and nanosilver up-regulation of enzymatic and non-enzymatic antioxidant activity and decreasing ROS content was observed which eventually resulted in extension of post harvest shelf life.

Our result indicated significant decrease in chlorophyll content in distilled water preserved leaves with increase in days of preservation. Decrease in chlorophyll content directly links with less chlorophyll biosynthesis^8^ due to the activation of the enzyme chlorophyllase^32^. We observed significant negative correlation (p ≤ 0.01) between chlorophyll degradation and generation of ROS. With increase in days of preservation, excessive accumulation of O_2_^•−^ and H_2_O_2_, generates oxidative stress. The major site for ROS generation in leaves was chloroplast^33^. During senescence photosynthetic efficiency decreases, as a consequence CO_2_ fixation reduces and ROS generation increases using the unutilized energy of the chloroplast^34^. It was reported that senescence causes chloroplast isolation and generation of O_2_^•−^ through reduction of O_2_ via photosynthetic electron transport chain^35^ and consequently by the action of the enzyme superoxide dismutase, the generated O_2_^•−^ get converted into H_2_O_2_^36^. Generated ROS was capable of causing oxidative damage to proteins, carbohydrates, lipids, and nucleic acids, while excessive ROS accumulation leads to programme cell death^37,38^. O_2_^•−^, H_2_O_2_ generation in our study showed direct correlation with the increase in content of MDA. MDA is considered as an indicator of stress^39^ associated with cell and organelle damage^40^. Multiple regression analysis of positive and negative correlated parameters associated with chlorophyll (Table 1) content indicated the major involvement of MDA during stress in chlorophyll degradation. Our outcome was supported by the earlier works, indicating increase in lipid peroxidation causes degradation of chlorophyll^41,42^. Rajinder *et al*.^43^ reported that decrease in chlorophyll content during leaf senescence was due to the lipid peroxidation of chloroplast membrane. However leaves preserved in NS solution showed significant (p ≤ 0.05) retention of chlorophyll content than distilled water set. Our result suggest that NS and SN both acts as an effective preservative by preventing ROS generation, lipid peroxidation and retention of chlorophyll content. In comparison to distilled water, SN showed high efficacy in nullifying generation H_2_O_2_ (p ≤ 0.05), while NS was proven to be more effective preservative showing its potential in checking O_2_^•−^, and H_2_O_2_ generation, and lipid peroxidation (p ≤ 0.05). Prevention of ROS generation helps in preventing plastid membrane peroxidation and thus maintaining chlorophyll content, extending the shelf life.

**Table 1:** Statistics summary of multiple regression analysis keeping chlorophyll constant.

Model	R	R Square	Adjusted R Square	Std. Error of the Estimate	Durbin-Watson
1	0.988^a^	0.976	0.974	0.032820	
2	0.996^b^	0.991	0.989	0.021129	
3	0.999^c^	0.998	0.997	0.011702	2.524

^a^Predictors: (Constant), **MDA**.

^b^Predictors: (Constant), MDA, SOD.

^c^Predictors: (Constant), MDA, SOD, FLAVANOL.

^d^Dependent Variable: TOTAL CHLOROPHYLL.

Leaves preserved in NS and SN displays appropriate oxidative stress tolerance strategy by enhancing antioxidant enzymological activity. SOD converts O_2_^•−^ into H_2_O_2_^13^, present study demonstrated gradual increase in SOD in leaves preserved with NS solution, while reversed trend was observed in distilled water preserved leaves. CAT, APX and GPOX are primarily synthesized by the plant for scavenging excess H_2_O_2_ produced by the activity of SOD^14^. Current study showed an increasing trend of CAT, APX and GPOX activity with increase in days of preservation in NS and SN set. While CAT activity increases in leaves preserved in NS and SN up to 6DS, in distilled water preserved leaves CAT activity fails to enhance during preservation, indicating its inability to nullify the toxic effect of H_2_O_2_. Similar results were obtained by application of nitrogenous compound on rose and *Chrysanthemum* cut flower^9,31^. Moreover CAT activity showed direct correlation with chlorophyll content (p ≤ 0.05), indicating H_2_O_2_ detoxification by CAT activity helps in chlorophyll retention with increase in days of preservation. APX and GPOX activity also showed increasing trend till the last day of preservation in NS and SN set. In distilled water set APX activity increases up to 4DS after which rapid decrease in activity was noted, while GPOX activity was much higher than leaves preserved in NS and SN solution till the last day. It has been reported that if CAT fails to nullify H_2_O_2_, APX and GPOX activity increases as a compensatory mechanism^14^. In distilled water set as CAT activity fails to rise during preservation so probably by increasing APX and GPOX activity leaves tried to detoxify generated H_2_O_2_. Obtained result suggest that in distilled water preserved leaves APX fails to detoxify H_2_O_2_ from 4DS onwards after which only GPOX plays the role as a single protecting agent against H_2_O_2_. GSR and GST activity shoot up in distilled water set till 4DS indicating high stress accumulation which the leaves tries to nullify by activating its protective measures. GST was reported to counteract the different abiotic damage caused during stress^44^ and also protects cells against oxygen toxicity^45^. On the contrary GSR was a flavo-protein oxidoreductase which plays crucial role in reducing biotic stress by maintaining ascorbate and glutathione pool in reduced state inside the chloroplast^46^. GSR and GST activity gradually increases in leaves preserved with NS solution till the last day indicating the potential of NS solution in strengthening the biological tissue for withstanding both abiotic and biotic stress.

A high increase in glutathione concentration in leaves preserved with NS solution was observed from 4DS till 6DS after which decrease in concentration takes place while no such high increase takes place for leaves preserved in SN solution but a gradual increasing trend was maintained till last day, reflecting an attempt in minimizing oxidative stress. Leaves in distilled water set fails to increase the concentration of glutathione and thus faces early senescence due to accumulation of oxidative stress. Glutathione was a low molecular weight non-protein thiol that performs multiple functions in biosynthetic pathways, detoxification and antioxidant activities which the other thiols cannot perform^47^. The non-enzymatic antioxidant activity of glutathione resides in its ability to generate another non-enzymatic antioxidant ascorbic acid, by participating in ascorbate – glutathione cycle^48^. Ascorbic acid, was reported to exist mostly in reduced state inside chloroplast^49^ and can directly participate in scavenging O_2_^•−^ and H_2_O_2_ via APX activity^15^. Our study reports increased concentration of ascorbic acid in leaves preserved in NS and SN solution indicating their ability to detoxify free radicals by involving APX, reported earlier and thereby enhancing postharvest shelf life. In addition to the pool of ascorbate and glutathione, leaves in NS solution also maintained almost steady pool of carotenoids which bears significant difference with decreasing content in distilled water set (p ≤ 0.05). Carotenoids belong to the class of lipophilic antioxidants, synthesized in plastids and thus might play role in preventing ROS accumulation in chloroplast^16^.

Besides ascorbic acid, glutathione and carotenoids, phenolics (total phenol, orthodihydric phenol and flavonoid) are another group of non-enzymatic antioxidant actively participates in stress shielding mechanism. The prime defensive function of polyphenols resides in lipid free radical scavenging activity^48^. Polyphenols by complexing metal ions prevents metal ion mediated conversion of O_2_^•−^ and H_2_O_2_ in to highly reactive hydroxyl radical and thereby preventing DNA damage^50^ and thus allowing continuous synthesis of compounds through enzymatic mechanism that are involved in enhancing shelf life. Our study reflects almost similar trend as describe above, with increase in days of preservation phenol and orthodihydric phenol content increases in leaves present in preservative solution reflecting there potentiality to overcome stress. Result of distilled water preserved leaves also reflects an increasing trend but the extent of increase in comparison to NS and SN was much less. Flavonoid content does not show any significant change with increasing days, however in distilled water set from 4DS onward a decreasing trend was observed explaining inability to overcome generated stress.

Free radical scavenging and metal chelating activity was measured for determining the potential of secondary metabolites in preventing stress caused by free radicals and metal ions, described above. It was observed that with increase in days of preservation ABTS^+^, DPPH, SO and NO scavenging activity increases in leaves preserved in NS and SN solution, on contradictory in distilled water set their activity decreases. Biogenic silver nanoparticles were reported to show high activity towards scavenging free radicals^51,52^ and chelating metal ions^53^. It seems that the scavenging potential of NS, as well as SN protects leaf tissue from the intracellular harmful effect of free radicals. Because of gradual deterioration at tissue level, secreted secondary metabolites oozes out from leaves preserved in distilled water showing high metal chelating activity than leaves preserved in NS and SN solution.

At post harvest stage, significant (p ≤ 0.05) retention of total protein content was recorded in leaves preserved in NS and SN solution. Retention of total and reducing sugar in NS set was also recorded to be significant (p ≤ 0.05). Post harvest shelf life was effected by number of factors and among them water relationship and balance plays a key role^54^. Water relation was interrupted mainly due to microbial proliferation, blocking the cut end and thereby preventing water channel^55,56^. NS was reported to enhance post harvest shelf life of flowering twigs at low concentration by decreasing microbial load^29,57^. It has also been reported that both SN and NS acts as an effective preservatives, but water conduction rate was high when NS was used as preservative^58^. Thus NS and SN by retaining the concentration of primary metabolite in preserved leaves to optimum level, allowing the synthesis of defensive enzymes needed to overcome the generated stress. We observed increased level of proline in all the preservative sets indicating activation of ROS scavenging mechanism^59^ and thereby stabilizing cellular proteins and membrane integrity^60^.

The production of good quality cocoon crop directly depends on quality of *M. alba* leaves provided during rearing phase^61^. Silkworm bears a special ability of converting ingested leaf protein into silk protein. Variation on leaf nutritional level puts significant impact on cocoon production both qualitatively and quantitatively^62^. Protein and carbohydrate are the two most important constituents of *M. alba* leaves playing crucial role in silkworm rearing. While leaf protein was essential for larval growth, silk gland development, and cocoon characters, carbohydrate serves as major energy source during accumulation and secretion of silk fibre from silk gland^63^. Our rearing records suggests that GI of larvae fed with leaves preserved in NS and SN solution remains almost same as that of larvae fed with fresh leaves. While significant (p ≤ 0.05) decrease in GI was observed when fed with leaves of distilled water set. Larval growth depends on protein content of the leaves, and retention of GI indicates retention of protein in leaves preserved in preservative solution. Multiple regression analysis of significantly correlated parameters associated with protein content (Table 2) in preserved leaves indicates greater the superoxide scavenging activity less was the ROS mediated protein oxidation and thus more was the native protein content in the leaves. ERR (%) was best observed in NS preserved leaves and thus indicating high viability and low mortality. Low ERR in larvae fed with distilled water preserved leaves describes their inability for cocoon production and thus high mortality rate. Productivity can be described in terms of three most important commercial parameters viz. cocoon weight, shell weight and shell ratio^63^. Larvae fed with NS preserved leaves showed best cocoon weight, shell weight and shell ration than larvae fed with leaves preserved with SN and distilled water. Such result was displayed might be due to more ingestion, digestion and conversion ratio of larvae fed with NS preserved leaves, demonstrating the ability of nanosilver solution as an effective preservative.

**Table 2:** Statistics summary of multiple regression analysis keeping protein constant.

Model	R	R Square	Adjusted R Square	Std. Error of the Estimate	Durbin-Watson
1	0.995^a^	0.991	0.990	1.212853	
2	0.997^b^	0.994	0.993	1.020261	1.843

^a^Predictors: (Constant), **SUPEROXIDE**.

^b^Predictors: (Constant), SUPEROXIDE, MDA.

^c^Dependent Variable: TOTAL PROTEIN.

The above experiments and explanations suggest that silver nanoparticles synthesized using *M. alba* leaf extract acts as an effective preservative in extending the shelf life of *M. alba* leaves at postharvest stage. Thus the specification of formed NS becomes an important criterion for leaf preservation. Synthesized NS showed SPR band at ~441 nm confirming the presence of nanosilver, as SPR spectra of silver nanoparticles appears in the wavelength range of 400–500 nm^64^. The synthesized nanoparticles are spherical in shape with more than 50% particles are in monodispersed phase and average particle size of ~14 nm representing high bioactivity, as bioactivity was inversely proportional to the size of nanoparticles^65–67^. Probably because of these bioactive nature biosynthesized silver nanoparticles able to retain the shelf life of *M. alba* leaves at postharvest stage. FT–IR spectra showed the involvement of proteins, amino acid, short peptides, carbohydrates and secondary metabolites like phenol as capping, stabilizing and bio-reducing agents^68^. The deviation in peak position between plant extract and nanosilver was the indication of bioreduction and was another confirming module of nanosilver formation^69^. From XRD peak at 38.312° corresponds to (111) Bragg’s reflection plane found to be most intensified peak, corresponding to this peak 10.071 nm crystalline size was obtained according to Debye-Scherrer’s equation and inter planar spacing from Bragg’s law was obtained to be 2.347 Å. XRD result also matches with standard JCPDS library file no: 04-0783 confirming face centered cubic structure of NS. Crystalline nature was further validated by SAED pattern, confirming orientation of nanocrystals around (111) plane.

## Conclusions

At postharvest stage of preservation, *M. alba* leaves exhibited decrease in chlorophyll, protein and carbohydrate concentration, accumulation of free radicals including ROS causing oxidative damage, lipid peroxidation and thus imposing changes in rearing parameters in the present study. Leaves preserved in NS and SN solution showed two line of defensive strategy; first the activation and upregulation of antioxidant enzymatic (SOD, CAT, APX, GPOX, GSR, GST) and non-enzymatic antioxidant (ascorbic acid, glutathione and carotenoids) activities; and secondly enhancement in production of secondary metabolites involved in free radical scavenging activity. These metabolic alterations allow the leaves at postharvest stage to withstand and extend shelf life. Leaves preserved in NS solution exhibits greater retention of photosynthetic pigments and other primary metabolites, probably due to greater ability to scavenge ROS both by enzymatic and non-enzymatic pathways. Moreover, feeding leaves preserved in NS solution showed better growth and cocoon parameters, indicating the absence of any adverse toxic effects. Thus it may be concluded that phytosynthesized silver nanoparticles at 6 ppm concentration bears the potentiality to extent the shelf life of *Morus alba* L. leaves. Our finding may regain the interest of landless farmers towards this wide field of application as they can undergo the process of rearing for some days by purchasing the leaves once.

## Materials and Methods

### Plant sample and experimental system

The experiment was carried out at Department of Botany, University of North Bengal, India (26°42′44.11″ N and 88°29′40.09″ E) during April – September of 2017–2018 and 2018–2019. The most widely grown and used cultivar of *Morus alba* in this region, namely S1 cultivar was used as experimental material and were collected from Matigara Sericulture Complex, Siliguri, India (26°70′40″ N and 88°35′37″ E) during daytime between 6 and 7 am, for maintaining fresh texture of leaves. Immature, unhealthy and diseased leaves were screened out for maintaining uniform experimental condition. Leaves were preserved up to 7 days in preservative solutions and during preservation perforated zipper bags were used for retaining moisture content. During experimental process three solutions were used as preservatives viz. phyto-synthesized silver nanoparticles (NS) containing 6 ppm colloidal silver, silver nitrate (SN) solution bearing 6 ppm silver ion which serves as positive control and distilled water serving as negative control; all the solutions were maintained at neutral pH (pH 7). Prior to preservation fine oblique section under water was made to the petiole of the leaves for maintaining intact water column. The experimental setup was kept at 25 °C maintaining light intensity of 260–270 lux and 40% humidity. Preserved leaves were collected at an interval of 1, 4, 6 and 7DS for evaluating the potentiality of preservative solutions in extending shelf life through chemical parameters with respect to fresh leaves.

### Phyto-synthesis of silver nanoparticles and its characterization

For phyto-synthesis 5 ml distilled water refluxed *M. alba* leaf extract (10 g leaf in 100 ml distilled water) was added to 45 ml 10^−3^ M silver nitrate solution (Sigma Aldrich) with continuous stirring in magnetic stirrer for 10 min. The colour of the solution gradually turns yellowish and finally to blackish brown within 20 min span, indicating nano formation^70^.

Silver nanoparticles formation was recorded in UV – Vis Spectrophotometer (Systronics 2201) as a function of wavelength ranges between 300–800 nm. The size and shape of nanoparticles was studied through TEM micrographic (Philips CM 200) operating at an accelerating voltage of 120 kV. The possible involvement of functional groups in nano formation was predicted through FT – IR analysis at a wavelength range of 500–4000 nm using potassium bromide pellet technique. The crystallinity of nanoparticles was determined through X-ray diffraction study (Burker ASX D8). The sample was Cu Kα radiated at an operational speed of 5° sec^−1^, keeping current, voltage, and wavelength constant at 35 mA, 40 kV, and 1.5 Å respectively.

### Determination of total chlorophyll, total soluble protein, free proline, total soluble sugar, and reducing sugar concentrations

Total chlorophyll content in preserved leaves was determined by Arnon method^71^. For extraction, 1 g leaf sample was homogenized with 20 ml of 80% (v/v) acetone and was centrifuged at 10,000 × g for 5 min at 25 °C. Obtained residue was re-extracted twice for complete extraction of pigments. The UV-Vis spectrophotometer (Systronics 2201) reading of the supernatants was taken at 663, and 645 nm for estimating chlorophyll content.

Total protein content was estimated following the method of Lowry *et al*.^72^. For extraction 0.5 g frozen leaf samples were crushed using sodium phosphate buffer and centrifuged at 5,000 × g at −10 °C for 5 min. To the supernatant 5 ml alkaline copper solution and 0.5 ml Folin-Ciocalteu reagent was added leading to the development of blue colour complex. The absorbance was measured at 660 nm after 30 min of incubation and was standardized using bovine serum albumin for determining the protein content. Free proline content was estimated according to the method described by Bares *et al*.^73^. For extraction 0.5 g leaf sample was homogenized in 10 ml 3% sulfosalicylic acid and estimation was done at 520 nm of the upper toluene layer isolated from the reaction mixture containing 1 ml extract, 2 ml acid ninhydrin, 2 ml glacial acetic acid, and 4 ml toluene.

Total soluble sugar and reducing sugar content was determined spectrophotometrically following the methods of Sadasivam and Manickam^74^. Using mortar and pestle 0.1 g of leaf samples were extracted twice with 80% hot ethanol, 5 ml each time. The ethanol present in the supernatant was heat evaporated and the final volume was made up to 10 ml by distilled water. Total soluble sugar was determined using anthrone reagent at 620 nm by incubating in a boiling water bath for 8 min. Reducing sugar was estimated at 510 nm using dinitrosalicyclic acid and 40% Rochelle salt solution.

### Determination of reactive oxygen species and lipid peroxidation accumulation

Accumulation of reactive oxygen species was evaluated by estimating hydrogen peroxide (H_2_O_2_) and superoxide (O_2_^•−^) content following the method prescribed by Loreto and Velikova^75^ and Elstner and Heupel^76^ respectively. H_2_O_2_ was estimated by homogenizing 0.5 g leaf sample in 1% (w/v) tri-chloroacetic acid (TCA) and centrifuging at 10,000 × g for 10 min at 4 °C. The obtained supernatant was spectrophotometrically measured at 390 nm after reacting with 10 mM phosphate buffer (pH 7.0) and 1 M potassium iodide. Superoxide content was estimated by homogenizing 1 g leaf samples in 65 mM potassium phosphate buffer (pH 7.8) and centrifuging at 5,000 × g for 10 min. The supernatant was reacted with 65 mM potassium phosphate buffer (pH 7.8) and 10 mM hydroxylamine hydrochloride, and incubated for 15 min at 25 °C. Then 17 mM sulfanilamide and 7 mM α-anaphthylamine were added and absorbance was recorded at 530 nm. Extend of lipid peroxidation was determined by measuring MDA contents following the method of Davenport *et al*.^77^. Leaf samples (0.2 g) were homogenized in 2 ml 0.5% (w/v) TCA and centrifuged at 10,000 × g for 10 min at 4 °C. The reaction mixture containing 2 ml supernatant and 2 ml 0.67% thiobarbituric acid (TBA) was incubated for 30 min at 95 °C, followed by ice water treatment to stop the reaction. The absorbance of the supernatant was recorded at 450, 532, 600 nm and MDA content was calculated using the following formula, MDA (µmol g^−1^) = [6.45 (A532 – A600) − 0.56 A450] × Vt/W, where Vt = 0.0021 and W = 0.2 g.

### Determination of free radical scavenging activities, and metal chelating activity

2,2-diphenyl-1-picrylhydrazyl (DPPH), 2,2-azino-bis 3-ethylbenzthiazoline-6-sulphonic acid (ABTS), superoxide (SO), nitric oxide (NO) scavenging activity, and metal chelating (MC) activity were determined following the method of Sidduraju *et al*.^78^, Li *et al*.^79^, Fu *et al*.^80^, Marcocci *et al*.^81^, and Dinis *et al*.^82^ respectively. Preserved leaf samples (1 g) were homogenized with 10 ml methanol and centrifuged at 10,000 × g for 10 min at 4 °C. The obtained supernatants were used as crude extract for the above cited assays.

DPPH scavenging activity was measured at 517 nm by using 0.2 ml methanolic extract against 2 ml DPPH. ABTS activity was determined by adding 2 ml ABTS to 1 ml extract and measuring the absorbance at 734 nm after 10 min incubation. For measuring superoxide activity absorbance was measured at 560 nm after 30 min illuminated light (4000 flux) incubation of the reaction mixture containing 1 ml nitroblue tetrazolium chloride, 1 ml nicotinamide adenine dinucleotide and 10 μl phenazine methosulphate. To measure nitric oxide scavenging activity 20 mM sodium nitroprusside, 0.5 ml phosphate buffer, and 3 ml Griess reagent was mixed with 0.5 ml extract and absorbance was measured at 540 nm after 30 min incubation. Metal chelating activity was measured at 562 nm by reacting methanolic extract with 2 mM FeCl_3_ and 5 mM Ferrozine. Scavenging activity was measured as percent inhibition using the following equation: % inhibition = [(A_0_ − A_1_)/A_0_] 100%, Where A_0_ and A_1_ are the absorbance of control and sample respectively. Antioxidant activity was expressed as concentration where 50% reduction in free radical takes place referred to as IC_50_ value.

### Determination of enzymatic, and non-enzymatic antioxidant activities

Enzymatic antioxidant activities were measured in terms of superoxide dismutase (SOD), catalase (CAT), glutathione disulfide reductase (GSR), glutathione peroxidase (GPOX), glutathione S-transferase (GST), and ascorbate peroxidase (APX) activity by following the methods given by Esfandiari *et al*.^83^, Hasanuzzaman *et al*.^84^, Chen and Asada^85^. SOD (EC: 1.15.1.1) activity was estimated by adding 0.05 ml enzyme extract to reaction mixture containing 200 mM methionine, 2.25 mM nitro-blue tetrazolium and 3 mM EDTA. Reaction was initiated by adding 60 µM riboflavin, incubated under light for 10 min, and absorbance was taken at 560 nm. Enzyme activity was expressed as quantity of enzyme reducing the absorbance of NBT by 50%. CAT (EC: 1.11.1.6) activity was determined by adding enzyme extract to a mixture containing 50 mM potassium phosphate buffer (pH 7.0), and 15 mM H_2_O_2_. The reaction was monitored at 240 nm for 1 min and the enzyme activity was expressed as unit (1unit = mmole of H_2_O_2_ reduced min^−1^ mg protein^−1^) using extinction coefficient of 39.4 M^−1^ cm^−1^. GSR (EC: 1.6.4.2) activity was recorded at 340 nm for 1 min by adding 1 mM glutathione disulfide to the reaction mixture containing 0.1 M potassium phosphate buffer (pH 7.8), 1 mM EDTA, 0.2 mM NADPH, and enzyme solution; the enzyme activity was expressed as unit (1unit = µmol (NADPH) min^−1^ mg protein^−1^) using extinction coefficient of 6.2 mM^−1^ cm^−1^. For measuring GPOX (EC: 1.11.1.9) activity reaction mixture contains 100 mM sodium phosphate buffer (pH 7.5), 1 mM EDTA, 1 mM sodium azide, 0.2 mM NADPH, 2 mM glutathione, 1 unit glutathione reductase, 0.6 mM H_2_O_2_, and 20 µl crude enzyme. Enzyme activity (unit) was recorded through oxidation of NADPH at 340 nm for 1 min and was calculated as µmol (NADPH) min^−1^ mg protein^−1^ using extinction coefficient of 6.62 mM^−1^ cm^−1^. GST (EC: 2.5.1.18) activity was expressed using extinction coefficient of 9.6 mM^−1^ cm^−1^ at 340 nm for 1 min from the reaction mixture containing enzyme solution in 100 mM tris-HCl (pH 6.5), 1.5 mM glutathione, and 1 mM 1-chloro-2,4-dinitrobenzene (CDNB). APX (EC: 1.11.1.11) activity was determined at 290 nm for 2 min by adding enzyme extract to 3 ml reaction mixture containing 50 mM potassium phosphate buffer (pH 7.0), 0.5 mM ascorbate, and 0.1 mM H_2_O_2_. One unit of APX was defined as amount of enzyme required to consume 1µmol of ascorbate min^−1^ mg protein^−1^.

Carotenoids content was determined by homogenizing 1 g samples in 80% acetone and centrifuging at 10,000 × g for 5 min at 25 °C. The absorbance of the supernatant was determined spectrophotometrically at 470, 645, and 663 nm^86^. For ascorbic acid determination, 0.5 g leaf samples were homogenized in 10% (w/v) trichloroacetic acid and centrifuged at 10,000 × g for 20 min at 25 °C. The obtained supernatant (0.5 ml) was incubated at 37 °C for 3 h after reacting with 2% 2, 4-dinitrophenyl hydrazine in 0.5 N H_2_SO_4_, and 10% thiourea. After incubation spectral reading was recorded at 520 nm^87^. Total glutathione content was determined by reacting 100 µl extract with 0.3 mM NADPH in 20 mM potassium phosphate buffer (pH 7.5) and 6 mM 5′-dithio-bis(2-nitrobenzoic acid). The reaction mixture was incubated at 25 °C for 3 min, and then 10 µl reduced glutathione was added, after the colour was developed absorbance was recorded at 412 nm^88^.

### Determination of polyphenol contents

For total phenol, ortho-dihydric phenol, and flavonoid estimation, 1 g leaf sample was ground with 80% ethanol and centrifuged at 10,000 × g for 20 min. The residue was re-extracted twice using 80% ethanol. The obtained supernatant was evaporated to dryness and the residue was dissolved with distilled water. Total phenol content was determined at 650 nm by adding 50% folin-ciocalteu reagent and 20% sodium carbonate to the extract following the method of Malick and Singh^89^, using gallic acid as standard. Orthodihydric phenol content was determined at 515 nm by adding 0.05 (N) HCl, Arnow’s reagent and 1 (N) NaOH to the aqueous extract following the method of Mahadevan and Sridhar^90^ using catechol as standards. Flavonoid content was determined at 510 nm following the method of Atanassova *et al*.^91^ by adding 5% NaNO_2_, 10% AlCl_3_ and 1 (M) NaOH to the aqueous extract and using quercetin as standard.

### Feeding experiment and rearing data collection

The overall rearing process was conducted under laboratory condition following the standard process of Krishnaswami *et al*.^92^. For rearing purpose healthy and disease free 5^th^ instar larvae (Nistari) were collected from Matigara Sericulture Complex. The larvae were distributed uniformly and randomly in bamboo made trays, each tray bearing 10 larvae. Larvae were supplemented with S1 genotype *M. alba* leaves. In one set freshly collected S1 leaves were given for feeding, while in other sets 1, 4, 6, and 7DS preserved leaves in distilled water, silver nitrate and nanosilver solution were given for feeding. During rearing process larval weight and mortality rate was recorded at regular interval. When the larvae started spinning they were left undisturbed. At the end of the trial, larval and cocoon parameters were calculated using following formulas:

$${\bf{G}}{\bf{r}}{\bf{o}}{\bf{w}}{\bf{t}}{\bf{h}}\,{\bf{i}}{\bf{n}}{\bf{d}}{\bf{e}}{\bf{x}}\,({\bf{G}}{\bf{I}})=\frac{{\rm{Final}}\,{\rm{weight}}\,{\rm{of}}\,{\rm{larvae}}\,({\rm{gm}})-{\rm{Initial}}\,{\rm{weight}}\,{\rm{of}}\,{\rm{larvae}}\,({\rm{gm}})}{{\rm{Initial}}\,{\rm{weight}}\,{\rm{of}}\,{\rm{larvae}}\,({\rm{gm}})}$$

$${\bf{S}}{\bf{i}}{\bf{n}}{\bf{g}}{\bf{l}}{\bf{e}}\,{\bf{c}}{\bf{o}}{\bf{c}}{\bf{o}}{\bf{o}}{\bf{n}}\,{\bf{w}}{\bf{e}}{\bf{i}}{\bf{g}}{\bf{h}}{\bf{t}}\,({\bf{S}}{\bf{C}}{\bf{W}})=\frac{{\rm{Weight}}\,{\rm{of}}\,10\,{\rm{male}}\,{\rm{cocoon}}\,({\rm{gm}})+{\rm{Weight}}\,{\rm{of}}\,10\,{\rm{female}}\,{\rm{cocoon}}\,({\rm{gm}})}{{\rm{Total}}\,{\rm{number}}\,{\rm{of}}\,{\rm{cocoon}}\,(20)}$$

$${\bf{S}}{\bf{i}}{\bf{n}}{\bf{g}}{\bf{l}}{\bf{e}}\,{\bf{s}}{\bf{h}}{\bf{e}}{\bf{l}}{\bf{l}}\,{\bf{w}}{\bf{e}}{\bf{i}}{\bf{g}}{\bf{h}}{\bf{t}}\,({\bf{S}}{\bf{S}}{\bf{W}})=\frac{{\rm{Weight}}\,{\rm{of}}\,10\,{\rm{male}}\,{\rm{shell}}\,({\rm{gm}})+{\rm{Weight}}\,{\rm{of}}\,10\,{\rm{female}}\,{\rm{shell}}\,({\rm{gm}})}{{\rm{Total}}\,{\rm{number}}\,{\rm{of}}\,{\rm{shell}}\,(20)}$$

$${\bf{S}}{\bf{h}}{\bf{e}}{\bf{l}}{\bf{l}}\,{\bf{r}}{\bf{a}}{\bf{t}}{\bf{i}}{\bf{o}}\,({\bf{S}}{\bf{R}}) \% =\frac{{\rm{Single}}\,{\rm{shell}}\,{\rm{weight}}\,({\rm{gm}})}{{\rm{Single}}\,{\rm{cocoon}}\,{\rm{weight}}\,({\rm{gm}})}\times 100$$

$${\bf{E}}{\bf{f}}{\bf{f}}{\bf{e}}{\bf{c}}{\bf{t}}{\bf{i}}{\bf{v}}{\bf{e}}\,{\bf{r}}{\bf{e}}{\bf{a}}{\bf{r}}{\bf{i}}{\bf{n}}{\bf{g}}\,{\bf{r}}{\bf{a}}{\bf{t}}{\bf{e}}\,({\bf{E}}{\bf{R}}{\bf{R}}) \% =\frac{{\rm{Total}}\,{\rm{number}}\,{\rm{of}}\,{\rm{cocoon}}\,{\rm{harvested}}}{{\rm{Total}}\,{\rm{number}}\,{\rm{of}}\,{\rm{larvae}}\,{\rm{brushed}}}\,\times 100$$

$${\bf{M}}{\bf{o}}{\bf{r}}{\bf{t}}{\bf{a}}{\bf{l}}{\bf{i}}{\bf{t}}{\bf{y}}\,{\bf{r}}{\bf{a}}{\bf{t}}{\bf{e}}\,({\bf{M}}{\bf{R}}) \% =\frac{{\rm{Number}}\,{\rm{of}}\,{\rm{death}}\,{\rm{larvae}}}{{\rm{Total}}\,{\rm{number}}\,{\rm{of}}\,{\rm{larvae}}}\times 100$$

### Data analysis

Using General Linear Model, two-way analysis of variance (ANOVA) was performed to calculate the effect of preservative solutions on preservation of *M. alba* leaves and on silk worm rearing (P ≤ 0.05) using SPSS statistical package (IBM SPSS Advanced Statistics 20.0). Bivariate correlations study using Pearson correlation coefficient was done to study interrelationship between different leaf metabolic parameters with rearing parameters (p ≤ 0.01 and 0.05) using SPSS. Multiple regression analysis was conducted using SPSS for determining the stress parameters that are involved in degradation of major primary metabolites. Figures were plotted using OriginPro 2018b software (b9.5.5.409).

## Supplementary information

Supplementary Information.
