# Supplementary material for: Use of biogenic silver nanoparticles in enhancing shelf life of Morus alba L. at post harvest stage
Source: Sci Rep. 2020 Jun 2;10:8923. doi: 10.1038/s41598-020-65953-7 (PMC7265373; doi:10.1038/s41598-020-65953-7)
Supplement: Supplementary file 1 — Supplementary Information. [file 41598_2020_65953_MOESM1_ESM.docx]

**Use of biogenic silver nanoparticles in enhancing shelf life of *Morus alba* L. at post harvest stage**

**Dipayan Das^1^ and Palash Mandal^2^***

^1^Senior Research Scholar, CSIR-UGC NET JRF, Plant Physiology and Pharmacognosy Research Laboratory, Department of Botany, University of North Bengal, Raja Rammohanpur, Siliguri, West Bengal, India – 734013

^2^Assistant Professor, Department of Botany, University of North Bengal, Raja Rammohanpur, Siliguri, West Bengal, India – 734013

*email: pmandalbotppprl@nbu.ac.in

**Supplementary Table 1.** General Linear Model representing significance level among preservative solutions with respect to different primary metabolite content at post harvest stage of preservation (Based on estimated marginal means, “*” denotes the mean difference is significant at the .05 level and “b” stands for adjustment for multiple comparisons according to Bonferroni.)

| **Dependent Variable** | | | **Mean Difference** | **Std. Error** | **Sig.^b^** | **95% Confidence Interval for Difference^b^** | |
| --- | --- | --- | --- | --- | --- | --- | --- |
|  |  |  |  |  |  | **Lower Bound** | **Upper Bound** |
| Total chlorophyll | Distilled water | Nano silver | -.334^*^ | .085 | .023 | -.613 | -.055 |
|  |  | Silver nitrate | -.260 | .085 | .066 | -.539 | .019 |
|  | Nano silver | Distilled water | .334^*^ | .085 | .023 | .055 | .613 |
|  |  | Silver nitrate | .074 | .085 | 1.000 | -.205 | .353 |
|  | Silver nitrate | Distilled water | .260 | .085 | .066 | -.019 | .539 |
|  |  | Nano silver | -.074 | .085 | 1.000 | -.353 | .205 |
| Total protein | Distilled water | Nano silver | -19.448^*^ | 5.215 | .029 | -36.592 | -2.304 |
|  |  | Silver nitrate | -17.615^*^ | 5.215 | .045 | -34.759 | -.471 |
|  | Nano silver | Distilled water | 19.448^*^ | 5.215 | .029 | 2.304 | 36.592 |
|  |  | Silver nitrate | 1.833 | 5.215 | 1.000 | -15.311 | 18.977 |
|  | Silver nitrate | Distilled water | 17.615^*^ | 5.215 | .045 | .471 | 34.759 |
|  |  | Nano silver | -1.833 | 5.215 | 1.000 | -18.977 | 15.311 |
| Total sugar | Distilled water | Nano silver | -10.042^*^ | 2.480 | .020 | -18.195 | -1.888 |
|  |  | Silver nitrate | -6.794 | 2.480 | .101 | -14.947 | 1.359 |
|  | Nano silver | Distilled water | 10.042^*^ | 2.480 | .020 | 1.888 | 18.195 |
|  |  | Silver nitrate | 3.248 | 2.480 | .715 | -4.906 | 11.401 |
|  | Silver nitrate | Distilled water | 6.794 | 2.480 | .101 | -1.359 | 14.947 |
|  |  | Nano silver | -3.248 | 2.480 | .715 | -11.401 | 4.906 |
| Reducing sugar | Distilled water | Nano silver | -1.477^*^ | .433 | .043 | -2.900 | -.055 |
|  |  | Silver nitrate | -1.357 | .433 | .060 | -2.779 | .065 |
|  | Nano silver | Distilled water | 1.477^*^ | .433 | .043 | .055 | 2.900 |
|  |  | Silver nitrate | .120 | .433 | 1.000 | -1.302 | 1.543 |
|  | Silver nitrate | Distilled water | 1.357 | .433 | .060 | -.065 | 2.779 |
|  |  | Nano silver | -.120 | .433 | 1.000 | -1.543 | 1.302 |
| Proline | Distilled water | Nano silver | .793 | .249 | .057 | -.027 | 1.612 |
|  |  | Silver nitrate | .649 | .249 | .122 | -.171 | 1.468 |
|  | Nano silver | Distilled water | -.793 | .249 | .057 | -1.612 | .027 |
|  |  | Silver nitrate | -.144 | .249 | 1.000 | -.963 | .676 |
|  | Silver nitrate | Distilled water | -.649 | .249 | .122 | -1.468 | .171 |
|  |  | Nano silver | .144 | .249 | 1.000 | -.676 | .963 |

**Supplementary Table 2.** General Linear Model representing significance level among preservative solutions with respect to stress accumulation at post harvest stage of preservation (Based on estimated marginal means, “*” denotes the mean difference is significant at the .05 level and “b” stands for adjustment for multiple comparisons according to Bonferroni.)

| **Dependent Variable** | | | **Mean Difference** | **Std. Error** | **Sig.^b^** | **95% Confidence Interval for Difference^b^** | |
| --- | --- | --- | --- | --- | --- | --- | --- |
|  |  |  |  |  |  | **Lower Bound** | **Upper Bound** |
| Hydrogen peroxide | Distilled water | Nano silver | 4.655^*^ | 1.135 | .019 | .923 | 8.388 |
|  |  | Silver nitrate | 3.822^*^ | 1.135 | .045 | .089 | 7.554 |
|  | Nano silver | Distilled water | -4.655^*^ | 1.135 | .019 | -8.388 | -.923 |
|  |  | Silver nitrate | -.833 | 1.135 | 1.000 | -4.566 | 2.899 |
|  | Silver nitrate | Distilled water | -3.822^*^ | 1.135 | .045 | -7.554 | -.089 |
|  |  | Nano silver | .833 | 1.135 | 1.000 | -2.899 | 4.566 |
| Superoxide | Distilled water | Nano silver | 4.186^*^ | 1.205 | .040 | .223 | 8.148 |
|  |  | Silver nitrate | 3.731 | 1.205 | .064 | -.231 | 7.693 |
|  | Nano silver | Distilled water | -4.186^*^ | 1.205 | .040 | -8.148 | -.223 |
|  |  | Silver nitrate | -.455 | 1.205 | 1.000 | -4.417 | 3.508 |
|  | Silver nitrate | Distilled water | -3.731 | 1.205 | .064 | -7.693 | .231 |
|  |  | Nano silver | .455 | 1.205 | 1.000 | -3.508 | 4.417 |
| MDA | Distilled water | Nano silver | 2.396^*^ | .664 | .034 | .213 | 4.579 |
|  |  | Silver nitrate | 1.962 | .664 | .076 | -.221 | 4.145 |
|  | Nano silver | Distilled water | -2.396^*^ | .664 | .034 | -4.579 | -.213 |
|  |  | Silver nitrate | -.433 | .664 | 1.000 | -2.616 | 1.750 |
|  | Silver nitrate | Distilled water | -1.962 | .664 | .076 | -4.145 | .221 |
|  |  | Nano silver | .433 | .664 | 1.000 | -1.750 | 2.616 |

**Supplementary Table 3.** General Linear Model representing significance level among preservative solutions with respect to antioxidant activity at post harvest stage of preservation (Based on estimated marginal means, “*” denotes the mean difference is significant at the .05 level and “b” stands for adjustment for multiple comparisons according to Bonferroni.)

| **Dependent Variable** | | | **Mean Difference** | **Std. Error** | **Sig.^b^** | **95% Confidence Interval for Difference^b^** | |
| --- | --- | --- | --- | --- | --- | --- | --- |
|  |  |  |  |  |  | **Lower Bound** | **Upper Bound** |
| ABTS | Distilled water | Nano silver | 2.562^*^ | .688 | .029 | .300 | 4.825 |
|  |  | Silver nitrate | 1.521 | .688 | .208 | -.742 | 3.783 |
|  | Nano silver | Distilled water | -2.562^*^ | .688 | .029 | -4.825 | -.300 |
|  |  | Silver nitrate | -1.042 | .688 | .543 | -3.304 | 1.221 |
|  | Silver nitrate | Distilled water | -1.521 | .688 | .208 | -3.783 | .742 |
|  |  | Nano silver | 1.042 | .688 | .543 | -1.221 | 3.304 |
| DPPH | Distilled water | Nano silver | 25.592^*^ | 7.149 | .035 | 2.090 | 49.093 |
|  |  | Silver nitrate | 24.876^*^ | 7.149 | .039 | 1.374 | 48.377 |
|  | Nano silver | Distilled water | -25.592^*^ | 7.149 | .035 | -49.093 | -2.090 |
|  |  | Silver nitrate | -.716 | 7.149 | 1.000 | -24.218 | 22.785 |
|  | Silver nitrate | Distilled water | -24.876^*^ | 7.149 | .039 | -48.377 | -1.374 |
|  |  | Nano silver | .716 | 7.149 | 1.000 | -22.785 | 24.218 |
| Superoxide  scavenging | Distilled water | Nano silver | 31.594 | 11.794 | .110 | -7.179 | 70.367 |
|  |  | Silver nitrate | 30.322 | 11.794 | .127 | -8.451 | 69.095 |
|  | Nano silver | Distilled water | -31.594 | 11.794 | .110 | -70.367 | 7.179 |
|  |  | Silver nitrate | -1.272 | 11.794 | 1.000 | -40.045 | 37.501 |
|  | Silver nitrate | Distilled water | -30.322 | 11.794 | .127 | -69.095 | 8.451 |
|  |  | Nano silver | 1.272 | 11.794 | 1.000 | -37.501 | 40.045 |
| Nitricoxide  scavenging | Distilled water | Nano silver | 6.627 | 2.625 | .135 | -2.004 | 15.257 |
|  |  | Silver nitrate | 8.245 | 2.625 | .060 | -.385 | 16.875 |
|  | Nano silver | Distilled water | -6.627 | 2.625 | .135 | -15.257 | 2.004 |
|  |  | Silver nitrate | 1.618 | 2.625 | 1.000 | -7.012 | 10.248 |
|  | Silver nitrate | Distilled water | -8.245 | 2.625 | .060 | -16.875 | .385 |
|  |  | Nano silver | -1.618 | 2.625 | 1.000 | -10.248 | 7.012 |
| Metal  chelating | Distilled water | Nano silver | 1.097 | .677 | .469 | -1.129 | 3.322 |
|  |  | Silver nitrate | -.210 | .677 | 1.000 | -2.435 | 2.016 |
|  | Nano silver | Distilled water | -1.097 | .677 | .469 | -3.322 | 1.129 |
|  |  | Silver nitrate | -1.306 | .677 | .306 | -3.532 | .919 |
|  | Silver nitrate | Distilled water | .210 | .677 | 1.000 | -2.016 | 2.435 |
|  |  | Nano silver | 1.306 | .677 | .306 | -.919 | 3.532 |

**Supplementary Table 4.** General Linear Model representing significance level among preservative solutions with respect to enzymatic antioxidant activity at post harvest stage of preservation (Based on estimated marginal means, “*” denotes the mean difference is significant at the .05 level and “b” stands for adjustment for multiple comparisons according to Bonferroni.)

| **Dependent Variable** | | | **Mean Difference** | **Std. Error** | **Sig.^b^** | **95% Confidence Interval for Difference^b^** | |
| --- | --- | --- | --- | --- | --- | --- | --- |
|  |  |  |  |  |  | **Lower Bound** | **Upper Bound** |
| CAT | Distilled water | Nano silver | -3.195 | 1.218 | .118 | -7.199 | .809 |
|  |  | Silver nitrate | -2.271 | 1.218 | .335 | -6.275 | 1.733 |
|  | Nano silver | Distilled water | 3.195 | 1.218 | .118 | -.809 | 7.199 |
|  |  | Silver nitrate | .924 | 1.218 | 1.000 | -3.081 | 4.928 |
|  | Silver nitrate | Distilled water | 2.271 | 1.218 | .335 | -1.733 | 6.275 |
|  |  | Nano silver | -.924 | 1.218 | 1.000 | -4.928 | 3.081 |
| GSR | Distilled water | Nano silver | .222 | 3.034 | 1.000 | -9.751 | 10.195 |
|  |  | Silver nitrate | .561 | 3.034 | 1.000 | -9.412 | 10.534 |
|  | Nano silver | Distilled water | -.222 | 3.034 | 1.000 | -10.195 | 9.751 |
|  |  | Silver nitrate | .339 | 3.034 | 1.000 | -9.634 | 10.312 |
|  | Silver nitrate | Distilled water | -.561 | 3.034 | 1.000 | -10.534 | 9.412 |
|  |  | Nano silver | -.339 | 3.034 | 1.000 | -10.312 | 9.634 |
| GPOX | Distilled water | Nano silver | 1.389 | .570 | .152 | -.483 | 3.262 |
|  |  | Silver nitrate | 1.520 | .570 | .111 | -.353 | 3.392 |
|  | Nano silver | Distilled water | -1.389 | .570 | .152 | -3.262 | .483 |
|  |  | Silver nitrate | .131 | .570 | 1.000 | -1.742 | 2.003 |
|  | Silver nitrate | Distilled water | -1.520 | .570 | .111 | -3.392 | .353 |
|  |  | Nano silver | -.131 | .570 | 1.000 | -2.003 | 1.742 |
| GST | Distilled water | Nano silver | -.838 | 5.794 | 1.000 | -19.886 | 18.210 |
|  |  | Silver nitrate | -2.510 | 5.794 | 1.000 | -21.558 | 16.538 |
|  | Nano silver | Distilled water | .838 | 5.794 | 1.000 | -18.210 | 19.886 |
|  |  | Silver nitrate | -1.672 | 5.794 | 1.000 | -20.720 | 17.376 |
|  | Silver nitrate | Distilled water | 2.510 | 5.794 | 1.000 | -16.538 | 21.558 |
|  |  | Nano silver | 1.672 | 5.794 | 1.000 | -17.376 | 20.720 |
| APX | Distilled water | Nano silver | -24.796 | 14.734 | .430 | -73.233 | 23.641 |
|  |  | Silver nitrate | -26.288 | 14.734 | .374 | -74.725 | 22.149 |
|  | Nano silver | Distilled water | 24.796 | 14.734 | .430 | -23.641 | 73.233 |
|  |  | Silver nitrate | -1.493 | 14.734 | 1.000 | -49.930 | 46.944 |
|  | Silver nitrate | Distilled water | 26.288 | 14.734 | .374 | -22.149 | 74.725 |
|  |  | Nano silver | 1.493 | 14.734 | 1.000 | -46.944 | 49.930 |
| SOD | Distilled water | Nano silver | -.163^*^ | .041 | .023 | -.299 | -.027 |
|  |  | Silver nitrate | -.121 | .041 | .078 | -.257 | .015 |
|  | Nano silver | Distilled water | .163^*^ | .041 | .023 | .027 | .299 |
|  |  | Silver nitrate | .042 | .041 | 1.000 | -.094 | .178 |
|  | Silver nitrate | Distilled water | .121 | .041 | .078 | -.015 | .257 |
|  |  | Nano silver | -.042 | .041 | 1.000 | -.178 | .094 |

**Supplementary Table 5.** General Linear Model representing significance level among preservative solutions with respect to non-enzymatic antioxidant activity at post harvest stage of preservation (Based on estimated marginal means, “*” denotes the mean difference is significant at the .05 level and “b” stands for adjustment for multiple comparisons according to Bonferroni.)

| Dependent Variable | | | Mean Difference (I-J) | Std. Error | Sig.^b^ | 95% Confidence Interval for Difference^b^ | |
| --- | --- | --- | --- | --- | --- | --- | --- |
|  |  |  |  |  |  | Lower Bound | Upper Bound |
| Carotenoids | Distilled water | Nano silver | -1.083^*^ | .250 | .015 | -1.906 | -.259 |
|  |  | Silver nitrate | -.737 | .250 | .078 | -1.560 | .087 |
|  | Nano silver | Distilled water | 1.083^*^ | .250 | .015 | .259 | 1.906 |
|  |  | Silver nitrate | .346 | .250 | .649 | -.477 | 1.169 |
|  | Silver nitrate | Distilled water | .737 | .250 | .078 | -.087 | 1.560 |
|  |  | Nano silver | -.346 | .250 | .649 | -1.169 | .477 |
| Ascorbic acid | Distilled water | Nano silver | -2.354^*^ | .651 | .033 | -4.494 | -.215 |
|  |  | Silver nitrate | -2.175^*^ | .651 | .047 | -4.314 | -.036 |
|  | Nano silver | Distilled water | 2.354^*^ | .651 | .033 | .215 | 4.494 |
|  |  | Silver nitrate | .179 | .651 | 1.000 | -1.960 | 2.318 |
|  | Silver nitrate | Distilled water | 2.175^*^ | .651 | .047 | .036 | 4.314 |
|  |  | Nano silver | -.179 | .651 | 1.000 | -2.318 | 1.960 |
| Total Glutathione | Distilled water | Nano silver | -3.238 | 1.140 | .089 | -6.985 | .509 |
|  |  | Silver nitrate | -2.085 | 1.140 | .351 | -5.832 | 1.662 |
|  | Nano silver | Distilled water | 3.238 | 1.140 | .089 | -.509 | 6.985 |
|  |  | Silver nitrate | 1.153 | 1.140 | 1.000 | -2.594 | 4.900 |
|  | Silver nitrate | Distilled water | 2.085 | 1.140 | .351 | -1.662 | 5.832 |
|  |  | Nano silver | -1.153 | 1.140 | 1.000 | -4.900 | 2.594 |

**Supplementary Table 6.** General Linear Model representing significance level among days of preservation with respect to secondary metabolites activity at post harvest stage of preservation (Based on estimated marginal means, “*” denotes the mean difference is significant at the .05 level and “b” stands for adjustment for multiple comparisons according to Bonferroni.)

| **Dependent Variable** | | | **Mean Difference** | **Std. Error** | **Sig.^b^** | **95% Confidence Interval for Difference^b^** | |
| --- | --- | --- | --- | --- | --- | --- | --- |
|  |  |  |  |  |  | **Lower Bound** | **Upper Bound** |
| Total Phenol | 1D | 4D | -1.372 | .711 | .610 | -4.118 | 1.373 |
|  |  | 6D | -2.232 | .711 | .120 | -4.978 | .513 |
|  |  | 7D | -3.121^*^ | .711 | .028 | -5.867 | -.375 |
|  | 4D | 1D | 1.372 | .711 | .610 | -1.373 | 4.118 |
|  |  | 6D | -.860 | .711 | 1.000 | -3.606 | 1.886 |
|  |  | 7D | -1.749 | .711 | .295 | -4.494 | .997 |
|  | 6D | 1D | 2.232 | .711 | .120 | -.513 | 4.978 |
|  |  | 4D | .860 | .711 | 1.000 | -1.886 | 3.606 |
|  |  | 7D | -.889 | .711 | 1.000 | -3.634 | 1.857 |
|  | 7D | 1D | 3.121^*^ | .711 | .028 | .375 | 5.867 |
|  |  | 4D | 1.749 | .711 | .295 | -.997 | 4.494 |
|  |  | 6D | .889 | .711 | 1.000 | -1.857 | 3.634 |
| Ortho Phenol | 1D | 4D | -.081 | .045 | .734 | -.255 | .093 |
|  |  | 6D | -.139 | .045 | .127 | -.313 | .034 |
|  |  | 7D | -.128 | .045 | .175 | -.302 | .046 |
|  | 4D | 1D | .081 | .045 | .734 | -.093 | .255 |
|  |  | 6D | -.059 | .045 | 1.000 | -.232 | .115 |
|  |  | 7D | -.047 | .045 | 1.000 | -.221 | .127 |
|  | 6D | 1D | .139 | .045 | .127 | -.034 | .313 |
|  |  | 4D | .059 | .045 | 1.000 | -.115 | .232 |
|  |  | 7D | .011 | .045 | 1.000 | -.163 | .185 |
|  | 7D | 1D | .128 | .045 | .175 | -.046 | .302 |
|  |  | 4D | .047 | .045 | 1.000 | -.127 | .221 |
|  |  | 6D | -.011 | .045 | 1.000 | -.185 | .163 |
| Flavonoid | 1D | 4D | -.408 | .427 | 1.000 | -2.058 | 1.242 |
|  |  | 6D | -.041 | .427 | 1.000 | -1.691 | 1.609 |
|  |  | 7D | .115 | .427 | 1.000 | -1.535 | 1.765 |
|  | 4D | 1D | .408 | .427 | 1.000 | -1.242 | 2.058 |
|  |  | 6D | .367 | .427 | 1.000 | -1.283 | 2.017 |
|  |  | 7D | .523 | .427 | 1.000 | -1.127 | 2.173 |
|  | 6D | 1D | .041 | .427 | 1.000 | -1.609 | 1.691 |
|  |  | 4D | -.367 | .427 | 1.000 | -2.017 | 1.283 |
|  |  | 7D | .156 | .427 | 1.000 | -1.494 | 1.806 |
|  | 7D | 1D | -.115 | .427 | 1.000 | -1.765 | 1.535 |
|  |  | 4D | -.523 | .427 | 1.000 | -2.173 | 1.127 |
|  |  | 6D | -.156 | .427 | 1.000 | -1.806 | 1.494 |

**Supplementary Table 7.** General Linear Model representing significance level among preservative solutions with respect to rearing parameters obtained by feeding larvae with preserved leaves (Based on estimated marginal means, “*” denotes the mean difference is significant at the .05 level and “b” stands for adjustment for multiple comparisons according to Bonferroni.)

| **Dependent Variable** | | | **Mean Difference (I-J)** | **Std. Error** | **Sig.^b^** | **95% Confidence Interval for Difference^b^** | |
| --- | --- | --- | --- | --- | --- | --- | --- |
|  |  |  |  |  |  | **Lower Bound** | **Upper Bound** |
| Growth Index | Distilled water | Nano silver | -.700^*^ | .200 | .039 | -1.359 | -.042 |
|  |  | Silver nitrate | -.674^*^ | .200 | .045 | -1.333 | -.016 |
|  | Nano silver | Distilled water | .700^*^ | .200 | .039 | .042 | 1.359 |
|  |  | Silver nitrate | .026 | .200 | 1.000 | -.633 | .685 |
|  | Silver nitrate | Distilled water | .674^*^ | .200 | .045 | .016 | 1.333 |
|  |  | Nano silver | -.026 | .200 | 1.000 | -.685 | .633 |
| Single Cocoon weight | Distilled water | Nano silver | -.768^*^ | .214 | .035 | -1.473 | -.063 |
|  |  | Silver nitrate | -.731^*^ | .214 | .043 | -1.436 | -.026 |
|  | Nano silver | Distilled water | .768^*^ | .214 | .035 | .063 | 1.473 |
|  |  | Silver nitrate | .037 | .214 | 1.000 | -.668 | .742 |
|  | Silver nitrate | Distilled water | .731^*^ | .214 | .043 | .026 | 1.436 |
|  |  | Nano silver | -.037 | .214 | 1.000 | -.742 | .668 |
| Single Shell weight | Distilled water | Nano silver | -.178^*^ | .047 | .027 | -.332 | -.024 |
|  |  | Silver nitrate | -.162^*^ | .047 | .040 | -.316 | -.008 |
|  | Nano silver | Distilled water | .178^*^ | .047 | .027 | .024 | .332 |
|  |  | Silver nitrate | .016 | .047 | 1.000 | -.138 | .170 |
|  | Silver nitrate | Distilled water | .162^*^ | .047 | .040 | .008 | .316 |
|  |  | Nano silver | -.016 | .047 | 1.000 | -.170 | .138 |
| Shell Ratio | Distilled water | Nano silver | -5.648^*^ | 1.491 | .027 | -10.548 | -.747 |
|  |  | Silver nitrate | -5.083^*^ | 1.491 | .043 | -9.983 | -.182 |
|  | Nano silver | Distilled water | 5.648^*^ | 1.491 | .027 | .747 | 10.548 |
|  |  | Silver nitrate | .565 | 1.491 | 1.000 | -4.335 | 5.465 |
|  | Silver nitrate | Distilled water | 5.083^*^ | 1.491 | .043 | .182 | 9.983 |
|  |  | Nano silver | -.565 | 1.491 | 1.000 | -5.465 | 4.335 |
| Effective Rate of Rearing | Distilled water | Nano silver | -45.834 | 14.169 | .053 | -92.415 | .748 |
|  |  | Silver nitrate | -38.334 | 14.169 | .106 | -84.915 | 8.248 |
|  | Nano silver | Distilled water | 45.834 | 14.169 | .053 | -.748 | 92.415 |
|  |  | Silver nitrate | 7.500 | 14.169 | 1.000 | -39.081 | 54.081 |
|  | Silver nitrate | Distilled water | 38.334 | 14.169 | .106 | -8.248 | 84.915 |
|  |  | Nano silver | -7.500 | 14.169 | 1.000 | -54.081 | 39.081 |
| Mortality Rate | Distilled water | Nano silver | 45.834 | 14.169 | .053 | -.748 | 92.415 |
|  |  | Silver nitrate | 38.334 | 14.169 | .106 | -8.248 | 84.915 |
|  | Nano silver | Distilled water | -45.834 | 14.169 | .053 | -92.415 | .748 |
|  |  | Silver nitrate | -7.500 | 14.169 | 1.000 | -54.081 | 39.081 |
|  | Silver nitrate | Distilled water | -38.334 | 14.169 | .106 | -84.915 | 8.248 |
|  |  | Nano silver | 7.500 | 14.169 | 1.000 | -39.081 | 54.081 |
